# Early-life peripheral infections reprogram retinal microglia and aggravate neovascular age-related macular degeneration in later life

**DOI:** 10.1172/JCI159757

**Published:** 2023-02-15

**Authors:** Masayuki Hata, Maki Hata, Elisabeth M.M.A. Andriessen, Rachel Juneau, Frédérique Pilon, Sergio Crespo-Garcia, Roberto Diaz-Marin, Vera Guber, Francois Binet, Frédérik Fournier, Manuel Buscarlet, Caroline Grou, Virginie Calderon, Emilie Heckel, Heather J. Melichar, Jean-Sebastien Joyal, Ariel M. Wilson, Przemyslaw Sapieha

**Affiliations:** 1 Department of Ophthalmology,; 2 Department of Biochemistry and Molecular Medicine, and; 3 Department of Biomedical Sciences, Maisonneuve-Rosemont Hospital Research Centre, University of Montreal, Montreal, Quebec, Canada.; 4 Bioinformatics Core Facility, Institut de recherches cliniques de Montréal, Montreal, Quebec, Canada.; 5 Department of Pediatrics, Ophthalmology, and Pharmacology, Centre Hospitalier Universitaire Ste-Justine Research Center, Montreal, Quebec, Canada.; 6 Department of Medicine, Maisonneuve-Rosemont Hospital Research Centre, University of Montreal, Montreal, Quebec, Canada.

**Keywords:** Ophthalmology, Cellular immune response, Endothelial cells

## Abstract

Pathological neovascularization in age-related macular degeneration (nvAMD) drives the principal cause of blindness in the elderly. While there is a robust genetic association between genes of innate immunity and AMD, genome-to-phenome relationships are low, suggesting a critical contribution of environmental triggers of disease. Possible insight comes from the observation that a past history of infection with pathogens such as *Chlamydia pneumoniae*, or other systemic inflammation, can predispose to nvAMD in later life. Using a mouse model of nvAMD with prior *C*. *pneumoniae* infection, endotoxin exposure, and genetic ablation of distinct immune cell populations, we demonstrated that peripheral infections elicited epigenetic reprogramming that led to a persistent memory state in retinal CX3CR1^+^ mononuclear phagocytes (MNPs). The immune imprinting persisted long after the initial inflammation had subsided and ultimately exacerbated choroidal neovascularization in a model of nvAMD. Single-cell assay for transposase-accessible chromatin sequencing (scATAC-seq) identified activating transcription factor 3 (ATF3) as a central mediator of retina-resident MNP reprogramming following peripheral inflammation. ATF3 polarized MNPs toward a reparative phenotype biased toward production of proangiogenic factors in response to subsequent injury. Therefore, a past history of bacterial endotoxin–induced inflammation can lead to immunological reprograming within CNS-resident MNPs and aggravate pathological angiogenesis in the aging retina.

## Graphical abstract

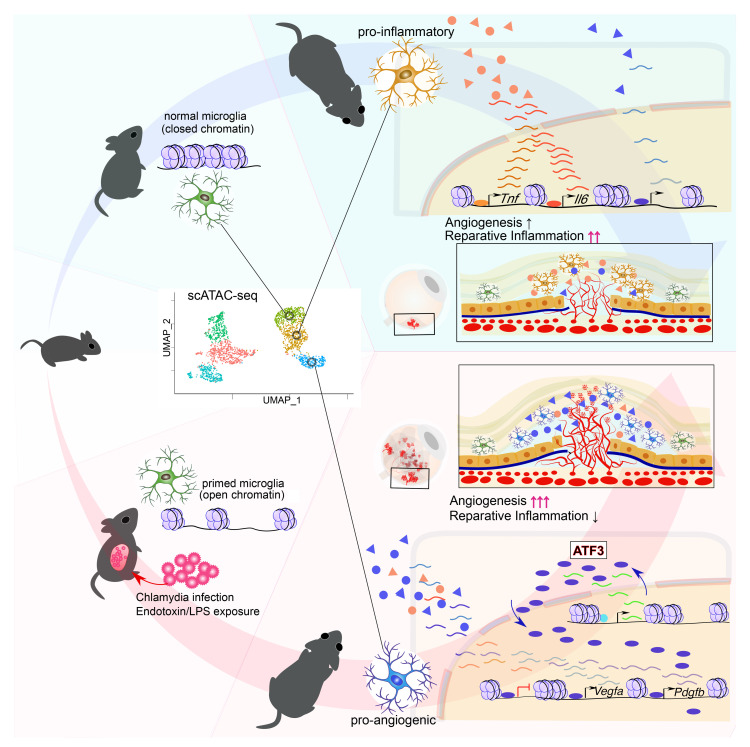

## Introduction

The search for genes that impact longevity has revealed low heritability of lifespan (1), highlighting instead the interplay of genetic and nongenetic factors (2). Genetics of conditions that influence health span are better defined; however, the association between phenome and genome remains relatively low in multivariable processes such as susceptibility to age-related diseases. There remains a need for deeper and more comprehensive mapping and integration of the multiple networks that influence gene function in disease (3). For instance, age-related macular degeneration (AMD) is a disease with identified genetic susceptibilities that profoundly impacts health span by causing irreversible blindness (4, 5). AMD is a leading cause of loss of sight worldwide, with the number of affected individuals rising to over 11 million in North America, and this is expected to double by 2050 (6). Genetic investigation identified a series of susceptibility loci for AMD in innate immunity–related genes, such as complement factor H (*CFH*), complement C2 (*C2*), complement C3 (*C3*), Toll-like receptor 4 (*TLR4*), and C-X3-C motif chemokine receptor 1 (*CX3CR1*). Importantly, polymorphisms in any susceptibility gene merely increase the incidence of AMD but do not guarantee its onset (7–10). This highlights the critical role of environmental factors, as well as previous life events, as disease triggers.

In patients suffering from the neovascular form of AMD (nvAMD), pathological neovascularization of choroidal blood vessels underneath the macula (choroidal neovascularization; CNV) rapidly compromises the central visual field. Over the past decade, standards of care for nvAMD have largely focused on targeting VEGF-A; yet, the long-term outcomes of patients on anti-VEGF therapies may be unfavorable due to macular atrophy (11). In addition, over 10% of patients show suboptimal response to anti–VEGF-A therapy (12, 13). Therefore, a deeper understanding of the mechanisms that instigate nvAMD may give rise to new strategies that stall onset of CNV or alter its trajectory.

While the etiology of AMD remains unclear, the innate immune response has been shown to play a central role in disease pathogenesis (14–16). Early stages of AMD are characterized by the accumulation of insoluble extracellular deposits, termed drusen, which contain immunogenic modified lipoproteins as well as complement factors that attract and activate resident retinal microglia and recruited macrophages (17). Activated phagocytes are found in the subretinal space of AMD patients (18). Furthermore, inadequate clearance of drusen by phagocytes triggers angiogenic responses that aggravate tissue damage and characterize late-stage nvAMD (16, 19). As a consequence, the reactivity of accumulating phagocytes is a driving force in photoreceptor demise and in disease manifestation (20), highlighting the importance of the innate immune system as a major driver of AMD pathophysiology (16).

Given both genetic links to the immune response and evidence of a local increase in innate immune cells in AMD, an intriguing disease-relevant association comes from the correlation between prior exposure to infectious agents such as *Chlamydia* *pneumoniae* and an increased risk of developing AMD (21–23). *C*. *pneumoniae* infection can rapidly cause macrophages to produce VEGF and migrate to target tissues (24, 25). However, the long-term impact of prior *C*. *pneumoniae* infection on innate immunity as well as the contributions of early-life immune events to AMD pathogenesis remain unknown. In the current study, we investigate whether exposure to peripheral immune stimuli can provoke sustained immune imprinting that alters inflammatory and angiogenic responses in the central nervous system (CNS) in later life and predispose to sight-threatening nvAMD.

## Results

### Prior infection with C. pneumoniae or systemic exposure to endotoxin aggravates pathological angiogenesis later in life.

**Figure 1:**
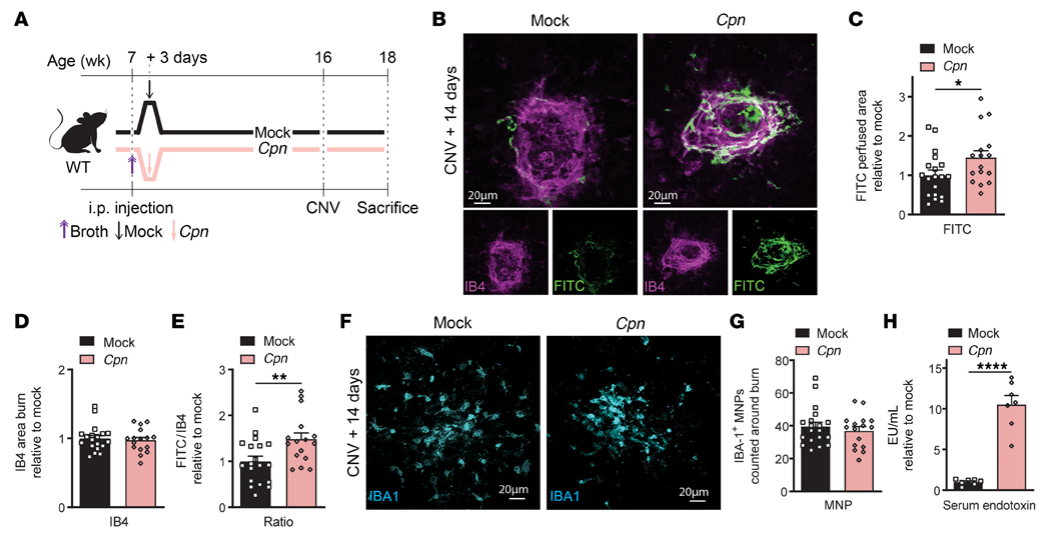
Prior peripheral infection predisposes to pathological angiogenesis in the retina. (**A**) Time course of *Chlamydia* *pneumoniae* (*Cpn*) or mock infections starting at 7 weeks. Laser-induced CNV occurred at 16 weeks, euthanasia at 18 weeks. (**B**) Confocal images of isolectin B4–stained (IB4-stained) laser burns with FITC-dextran–labeled CNVs. Scale bars: 20 μm. Quantification of (**C**) CNV area, (**D**) laser impact area, and (**E**) FITC/IB4 ratio per laser burn, all on day 14; *n* = 19 burns (Mock), *n* = 16 burns (*Cpn*). (**F**) Confocal images of mononuclear phagocytes (MNPs) stained for ionized calcium-binding adaptor molecule 1 (IBA1). Scale bars: 20 μm. (**G**) IBA1-positive MNP counts on day 14; *n* = 19 burns (Mock), *n* = 16 burns (*Cpn*). (**H**) Endotoxin levels (endotoxin units/mL) 5 days after infection; *n* = 7 per condition. Data are presented as mean ± SEM. Student’s unpaired *t* test (**C**–**E**, **G**, and **H**) was used. *P < 0.05; **P < 0.01; ****P < 0.0001.

To explore whether common infections occurring in early life predispose to pathological neovascularization in AMD in later life, we first developed a mouse model. We gave 7-week-old C57BL/6J male mice an intraperitoneal (i.p.) injection of thioglycollate broth followed 3 days later by inoculation with 5 × 10^5^ inclusion-forming units/mouse of *C*. *pneumoniae* (AR-39 strain) (Figure 1A). Chlamydia infection was confirmed by detection of chlamydial ribosomal DNA in peritoneal macrophages 3 days after *C*. *pneumoniae* inoculation (Supplemental Figure 1A; supplemental material available online with this article; https://doi.org/10.1172/JCI159757DS1). Body weight declined shortly after infection, but steadily returned to normal control levels by day 60 after infection (Supplemental Figure 1B). On day 60 after inoculation with *C*. *pneumoniae* (16 weeks of life), we induced CNV (Figure 1A). Fourteen days following laser burn, mice were perfused with FITC-dextran, the RPE-choroid-sclera complex flatmounted, and CNV area imaged by scanning laser confocal microscopy as previously described (26) (Figure 1B). Quantification of FITC-dextran–perfused neovessels revealed a robust, 50% increase in CNV area in mice previously infected with *C*. *pneumoniae* compared with control mice (Figure 1C). The average size of isolectin B4–labeled (IB4-labeled) lesions (comprising endothelial cells in neovascularization and choriocapillaris beneath the laser-burned Bruch’s membrane) did not differ between groups, suggesting that the observed effect was directly attributable to nascent perfused neovessels (Figure 1, D and E). Interestingly, the number of mononuclear phagocytes (MNPs; labeled with ionized calcium-binding adaptor molecule 1 [IBA1]) recruited to sites of injury in RPE-choroid-sclera complexes was comparable between *C*. *pneumoniae*–infected mice and control mice (Figure 1, F and G). Hence, a history of *C*. *pneumoniae* infection leads to persistent imprinting in later life that augments neovascularization in distal retinal tissue.

Given that lipopolysaccharide (LPS) is a major component of chlamydial endotoxin and that endotoxin levels were significantly elevated in the blood of *C*. *pneumoniae*–infected mice on day 5 after infection (Figure 1H), we next tested whether the primed state that exacerbates CNV can be recapitulated by exposing mice to peripheral LPS. Exposure to high concentrations of LPS has been shown to disrupt the blood-brain barrier (BBB) (27); hence, we first established the appropriate dosing paradigm for LPS to avoid severe systemic inflammation that could potentially directly influence the retina. We gave 7-week-old male C57BL/6J mice either 4 consecutive daily i.p. injections of LPS at 0.5 mg/kg (low-dose LPS) to mimic sustained infection with Gram-negative bacteria or a single bolus injection of LPS at 5 mg/kg (high-dose LPS) following 3 days of vehicle (PBS) injection. Control mice received 4 consecutive injections of vehicle (Supplemental Figure 1C). Twenty-four hours after the last i.p. injection of high-dose LPS, endotoxin was detected in serum and brain, but not in retina/choroid complexes (Supplemental Figure 1, D–F). Consistent with previous reports (28, 29), after i.p. injection of low-dose LPS groups, endotoxin was not detected in the brain or in retina/choroid complexes, but increased in serum (Supplemental Figure 1, D–F). High-dose LPS increased BBB permeability, and, in parallel, led to upregulation of proinflammatory genes *Il1b*, *Il6*, and *Tnf*, as well as the antiinflammatory gene *Tgfb1* in the retina/choroid complexes (Supplemental Figure 1, G–M). Conversely, low-dose LPS did not affect blood-retina barrier (BRB) and BBB integrities, and did not significantly influence the expression of inflammatory genes. Hence, injection of low-dose LPS was appropriate to mimic Gram-negative infection while avoiding vascular leakage and severe neuroinflammation in the retina. Together, these data convinced us to select an LPS dose of 0.5 mg/kg for our experimental paradigms given that peripheral exposure to this dose of LPS did not breach the BRB or directly provoke severe inflammation in the retina.

**Figure 2:**
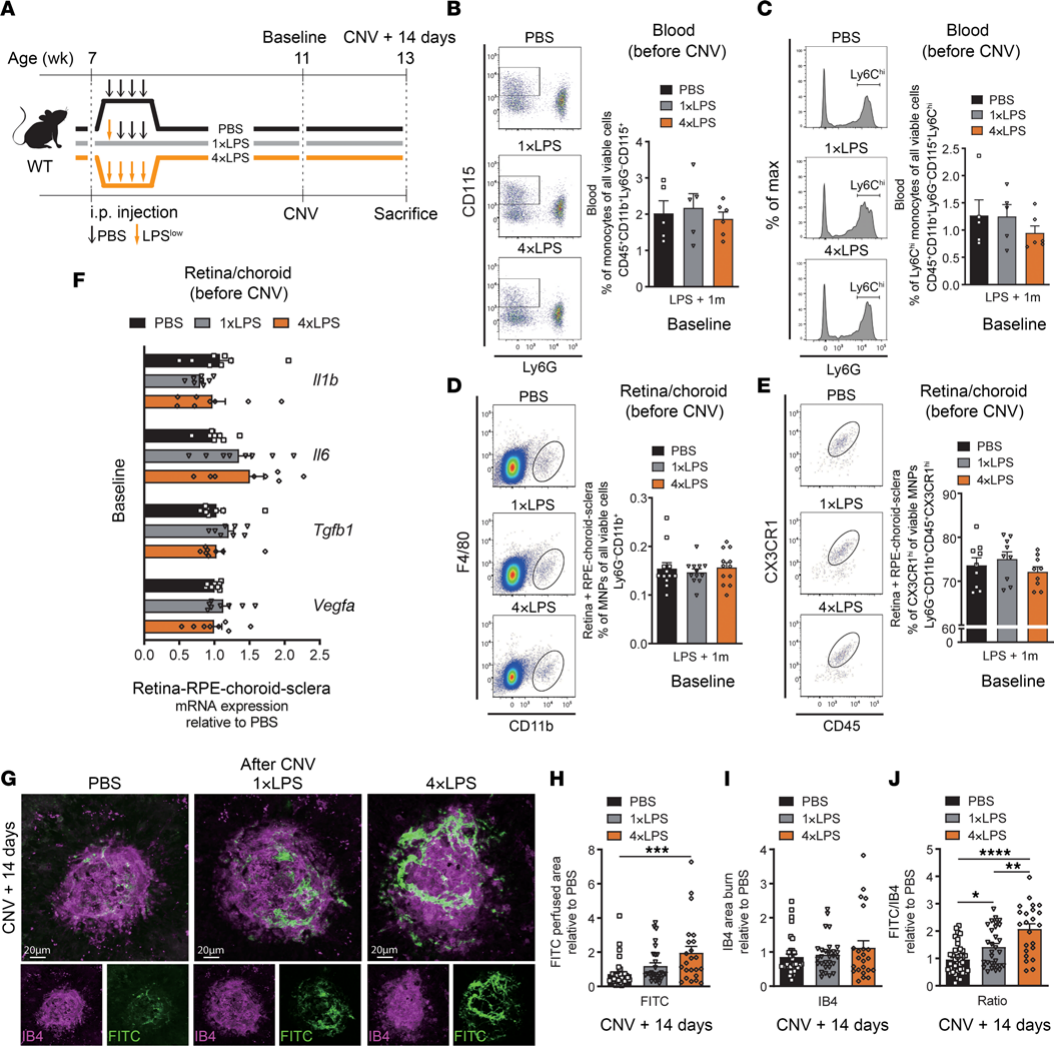
Prior systemic exposure to endotoxin predisposes to pathological angiogenesis in the retina. (**A**) Time course of peripheral LPS stimuli, where C57BL/6J mice received 1 low-dose injection of LPS (1×LPS), 4 daily injections of LPS (4×LPS), or 4 daily PBS injections (PBS) at 7 weeks old. Laser-induced CNV occurred at 11 weeks and euthanasia at 13 weeks. (**B**) Flow cytometry plots and percentage of viable blood monocytes in PBS (*n* = 5), 1×LPS (*n* = 5), and 4×LPS (*n* = 6) groups. (**C**) Plots and percentage of viable Ly6C^hi^ monocytes in PBS (*n* = 5), 1×LPS (*n* = 5), and 4×LPS (*n* = 6) groups. (**D**) Plots and percentage of viable MNPs in PBS, 1×LPS, and 4×LPS groups (*n* = 12 per condition). (**E**) Plots and percentage of viable CX3CR1^+^ microglia in PBS, 1×LPS, and 4×LPS groups (*n* = 9 per condition). (**F**) *Il1b*, *Il6*, *Tgfb1*, and *Vegfa* mRNA expression in retina/RPE-choroid-sclera complexes 4 weeks after LPS or PBS injections; *n* = 8 per condition. (**G**) CNV confocal imaging of IB4 and FITC-dextran from PBS, 1×LPS, and 4×LPS groups. Scale bars: 20 μm. Quantification of (**H**) CNV area, (**I**) laser impact area, and (**J**) FITC/IB4 ratio per laser burn on day 14; *n* = 39 burns (PBS), *n* = 29 burns (1×LPS), *n* = 23 burns (4×LPS). Data are presented as mean ± SEM. One-way ANOVA with Tukey’s multiple-comparison test (**B**–**F** and **H**–**J**) was used. *P < 0.05; **P < 0.01; ***P < 0.001; ****P < 0.0001.

We next treated mice according to 1 of 3 paradigms: (a) a single i.p. injection of LPS, followed by vehicle (PBS) injections for the subsequent 3 days (1×LPS group) to model acute exposure to Gram-negative bacteria; (b) a daily i.p. injection of LPS on 4 consecutive days (4×LPS group) to mimic sustained infection; and (c) a control group with 4 vehicle injections (PBS) (Figure 2A). Both groups treated with LPS showed a decrease in body weight soon after initial LPS injection that returned to control levels in less than 30 days (Supplemental Figure 1N). We first evaluated the effect of LPS treatment paradigms on levels of circulating monocytes. In blood, LPS-treated mice had a transient increase in the percentage of monocytes that returned to baseline levels within 1 month (Supplemental Figure 2, B and C, with gating in Supplemental Figure 2A). Four weeks following initial treatment with LPS, we confirmed that percentages of monocytes in blood, and more specifically Ly6C^hi^ monocytes (Figure 2, B and C), were comparable across groups. Similarly, the percentages of retinal/choroidal MNPs and CX3CR1^+^ MNPs did not vary during 1 month subsequent to LPS injection (Supplemental Figure 2, E–G; gating in Supplemental Figure 2D) and were comparable across all 3 experimental groups after 4 weeks with both LPS protocols (Figure 2, D and E). The results were the same when divided into retina and choroid (Supplemental Figure 2, H–K). Real-time qPCR (RT-qPCR) showed that expression of *Il1b*, *Il6*, *Tgfb1*, and *Vegfa* transcriptsin the retina/choroid complexes were comparable among the 3 groups (Figure 2F). Hence, these data indicate that initial systemic inflammation following both LPS injection paradigms had subsided without leaving signs of retinal inflammation 4 weeks after injection.

We then subjected mice to laser-induced CNV 4 weeks after LPS injection (when retinal numbers of MNPs were equal across groups) (Figure 2, A and D). Both LPS paradigms heightened CNV formation 14 days after laser burn compared with control mice (Figure 2, G–J). Notably, CNV was significantly more pronounced in mice subjected to repeated treatment with LPS (4×LPS) when compared with mice that received a single dose of LPS (Figure 2J). Collectively, these data demonstrate that a prior exposure to infectious agents, such as *C*. *pneumoniae*, or simply exposure to the bacterial endotoxin, primes the retina and exacerbates CNV in later life.

### CX3CR1^+^ retinal myeloid cells are primed through peripheral preconditioning by endotoxins and drive pathological angiogenesis.

Exposure to endotoxins such as LPS has been shown to prime myeloid cells, including circulating monocytes/macrophages and brain-resident macrophages, to respond to secondary inflammatory challenge in a process termed innate immune memory (29–33). Given that heightened CNV secondary to prior peripheral exposure to endotoxins occurred (Figure 2), we investigated whether past exposure to peripheral LPS can induce memory in retinal MNPs and may potentiate neovascularization in AMD.

**Figure 3:**
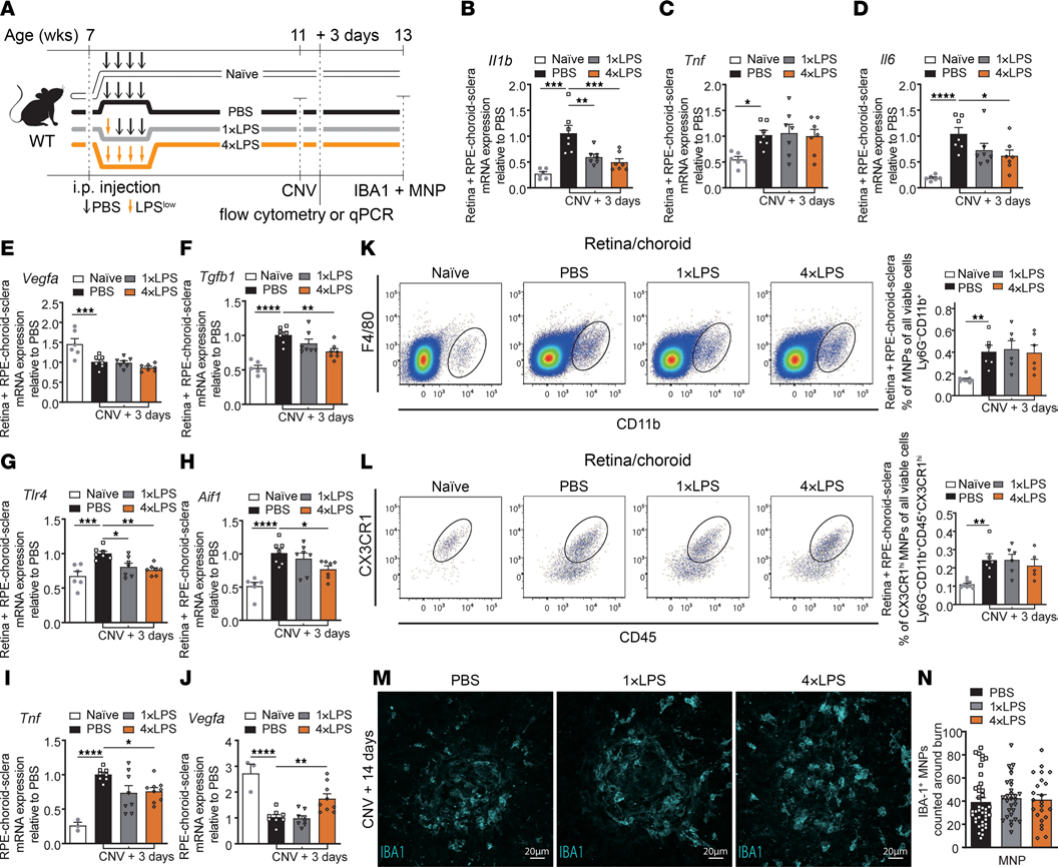
Prior systemic exposure to endotoxin mitigates retinal neuroinflammation. (**A**) Time course of peripheral LPS stimuli, where C57BL/6J mice received injections of LPS once (1×LPS), 4 daily injections of LPS (4×LPS), or 4 daily injections of PBS (PBS) at 7 weeks. Laser-induced CNV occurred at 11 weeks and euthanasia 3 days or 2 weeks later. Naive mice received PBS injections but no laser burns. (**B**–**H**) mRNA expression in retina/RPE-choroid-sclera complexes 3 days after CNV induction (PBS, 1×LPS, and 4×LPS) of *Il1b* (**B**), *Tnf* (**C**), *Il6* (**D**), *Vegfa* (**E**), *Tgfb1* (**F**), *Tlr4* (**G**), and *Aif1* (**H**): *n* = 6 (naive), *n* = 7 (PBS), *n* = 7 (1×LPS), *n* = 7 (4×LPS). (**I** and **J**) mRNA expression in RPE-choroid-sclera complexes 3 days after CNV induction (PBS, 1×LPS, and 4×LPS) or without CNV induction (naive) relative to PBS of (**I**) *Tnf* and (**J**) *Vegfa*: *n* = 3 (naive), *n* = 8 (PBS), *n* = 8 (1×LPS), *n* = 9 (4×LPS). (**K** and **L**) Flow cytometric analyses of whole retinas and RPE-choroid-sclera complexes 3 days after CNV induction. Plots and percentage of viable mononuclear phagocytes (MNPs) (**K**) and CX3CR1^+^ MNPs (**L**) in naive (*n* = 9), PBS (*n* = 6), 1×LPS (*n* = 6), and 4×LPS (*n* = 6) groups. (**M**) Confocal images of IBA1-stained MNPs on day 14 of PBS-, 1×LPS-, and 4×LPS-treated groups. Scale bars: 20 μm. (**N**) Number of IBA1-positive MNPs around laser impact area on day 14; *n* = 40 burns (PBS), *n* = 29 burns (1×LPS), *n* = 23 burns (4×LPS). Data are presented as mean ± SEM. One-way ANOVA with Tukey’s multiple-comparison test (**B**–**L** and **N**) was used. *P < 0.05; **P < 0.01; ***P < 0.001; ****P < 0.0001.

We exposed mice to either acute 1×LPS or protracted 4×LPS paradigms, induced CNV, and assessed the innate immune response (Figure 3A). Three days after laser-induced injury, levels of innate immunity–related genes were assessed in retina/choroid complexes by RT-qPCR (Figure 3, B–H). *Il1b*, *Il6, Tgfb1, Tlr4,* and *Aif1* expression was significantly decreased when compared with control PBS groups, suggesting an altered retinal immune response following conditioning with repeated systemic exposure to LPS (Figure 3, B, D, and F–H). Although *Tnf* and *Vegfa* expression was comparable in the retina/choroid complexes (Figure 3, C and E), decreased expression of *Tnf* and increased expression of *Vegfa* was noted for the 4×LPS group in the choroid (Figure 3, I and J), which might be related to the enhanced CNV observed in mice subjected to 4×LPS. Similarly to observations following *C*. *pneumoniae* infections (Figure 1, F and G), flow cytometry of retinas showed that while the number of MNPs increased following induction of CNV, and more specifically CX3CR1^+^ MNPs, at 3 days after laser-induced injury, proportions of CX3CR1^+^ MNPs did not vary between the PBS, 1×LPS, and 4×LPS groups (Figure 3, K and L). Immunofluorescent staining of flatmounted RPE-choroid-sclera complexes further confirmed that the number of MNPs, labeled with IBA1, recruited to sites of injury in RPE-choroid-sclera complexes did not differ between the 3 groups (Figure 3, M and N). These data suggest that past exposure to peripheral LPS can mitigate retinal neuroinflammation without affecting absolute numbers of lesion-associated MNPs. The dosing paradigm of 4×LPS was selected moving forward given that it presented a more robust preconditioned phenotype.

We next examined whether prior exposure to LPS could condition retinal myeloid cells and trigger innate immune memory that accounts for the heightened angiogenesis described above (Figure 2). We generated compound heterozygous mice carrying the *Cx3cr1*^CreER^ allele and the *R26* iDTR allele (*Cx3cr1*^CreER/+^:R26^iDTR/+^), in which Cre recombinase activation under the control of the *Cx3cr1* promoter can be induced by tamoxifen administration. This leads to deletion of the *loxP*-flanked STOP sequence and permits human diphtheria toxin receptor (DTR) expression in all CX3CR1^+^ myeloid cells after tamoxifen administration. DTR expression is almost exclusively restricted to long-lived microglia; it is not expressed in short-lived myeloid cells 1 month after tamoxifen administration (34, 35). CX3CR1 is preferentially expressed in myeloid cells, especially monocytes and tissue-resident macrophages (microglia in the retina) (Supplemental Figure 3, A and B). Following intravitreal administration of diphtheria toxin, ablation of local (retinal) myeloid cells was verified by flow cytometry; MNPs, especially CX3CR1^+^ cells, were decreased in the retina of *Cx3cr1*^CreER/+^:R26^iDTR/+^ mice, while CX3CR1^+^ MNPs, but not the total population of MNPs, were decreased in RPE-choroid-sclera complexes of *Cx3cr1*^CreER/+^:R26^iDTR/+^ mice (Supplemental Figure 3, C–H).

**Figure 4:**
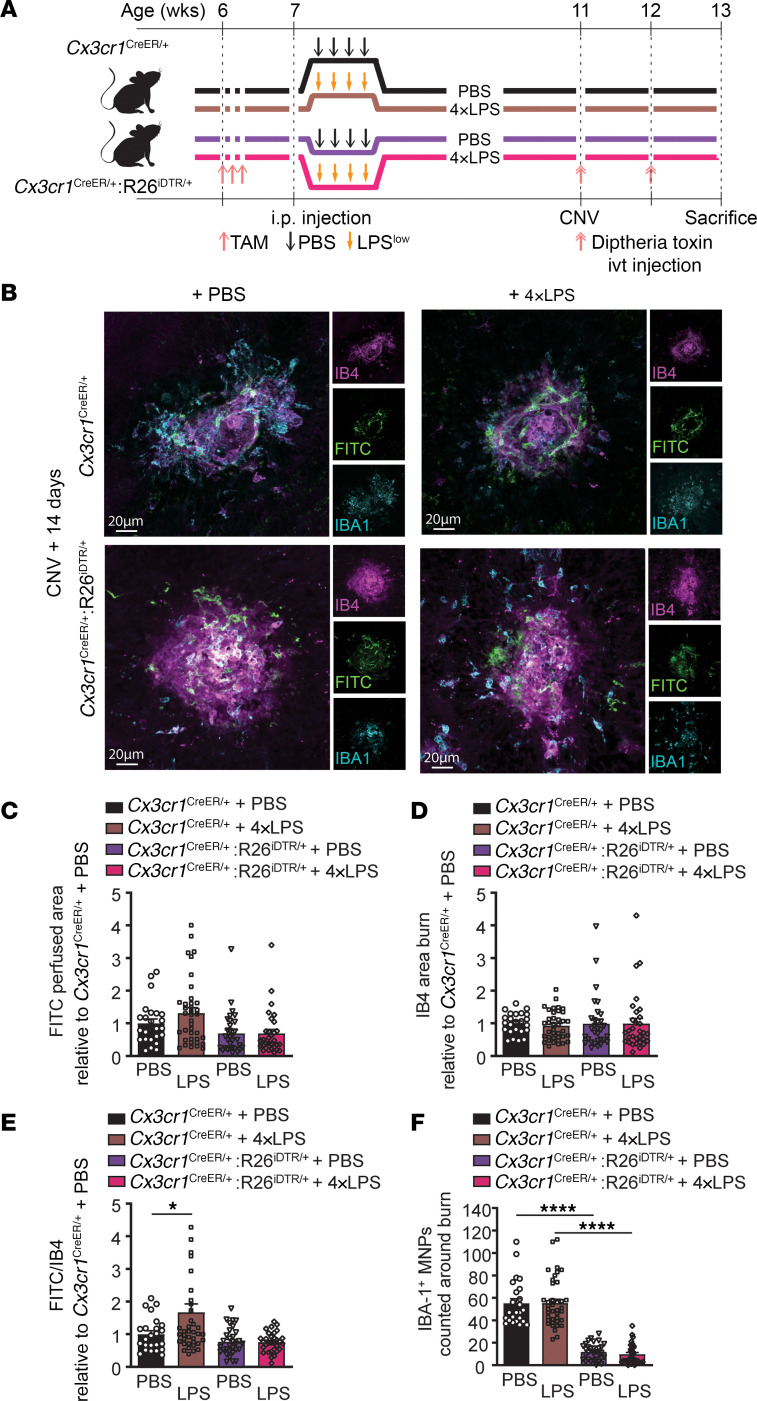
CX3CR1^+^ myeloid cells in the retina mediate proangiogenic memory after systemic exposure to LPS. (**A**) Time course of *Cx3cr1*^CreER/+^ and *Cx3cr1*^CreER/+^:R26^iDTR/+^ mice injected with 4×LPS or PBS at 7 weeks. For both *Cx3cr1*^CreER/+^ and *Cx3cr1*^CreER/+^:R26^iDTR/+^ mice, tamoxifen (TAM) was administered i.p. starting at 6 weeks and diphtheria toxin intravitreally (ivt) at week 11 and 12. Laser-induced CNV occurred at 11 weeks, euthanasia at week 13. (**B**) CNV confocal images of IB4, FITC-dextran, and IBA1 staining from *Cx3cr1*^CreER/+^ and *Cx3cr1*^CreER/+^:R26^iDTR/+^ mice with either 4×LPS or PBS injections. Scale bars: 20 μm. (**C**–**F**) Quantification of CNV area (**C**), IB4-stained laser impact area (**D**), FITC/IB4 ratio per laser burn (**E**), and number of IBA1-positive MNPs (**F**) on day 14; *n* = 24 burns (*Cx3cr1*^CreER/+^ + PBS), *n* = 38 burns (*Cx3cr1*^CreER/+^ + 4×LPS), *n* = 31 burns (*Cx3cr1*^CreER/+^:R26^iDTR/+^ + PBS), *n* = 31 burns (*Cx3cr1*^CreER/+^:R26^iDTR/+^ + 4×LPS). Data are presented as mean ± SEM. One-way ANOVA with Tukey’s multiple-comparison test (**C**–**F**) was used. *P < 0.05; ****P < 0.0001.

To determine whether exposure to peripheral endotoxin leads to innate immune imprinting, *Cx3cr1*^CreER/+^:R26^iDTR/+^ mice were given i.p. injections of tamoxifen for 3 consecutive days at 6 weeks of age and were treated i.p. with either 4×LPS or PBS 1 week later. Diphtheria toxin was administered locally to the vitreous at 11 and 12 weeks of age, with laser-induced CNV at 11 weeks of age (Figure 4A). Fourteen days following laser burn, CNV formation was assessed and the 4×LPS group showed an increase in CNV area when compared with the PBS control group in *Cx3cr1*^CreER/+^ mice with injections of tamoxifen and diphtheria toxin (Figure 4, B–E). Conversely, this effect was lost in *Cx3cr1*^CreER/+^:R26^iDTR/+^ mice with decreased numbers of lesion-associated MNPs (Figure 4, B–F), confirming that prior exposure to endotoxin leads to heightened pathological angiogenesis via retina-resident myeloid cells.

**Figure 5:**
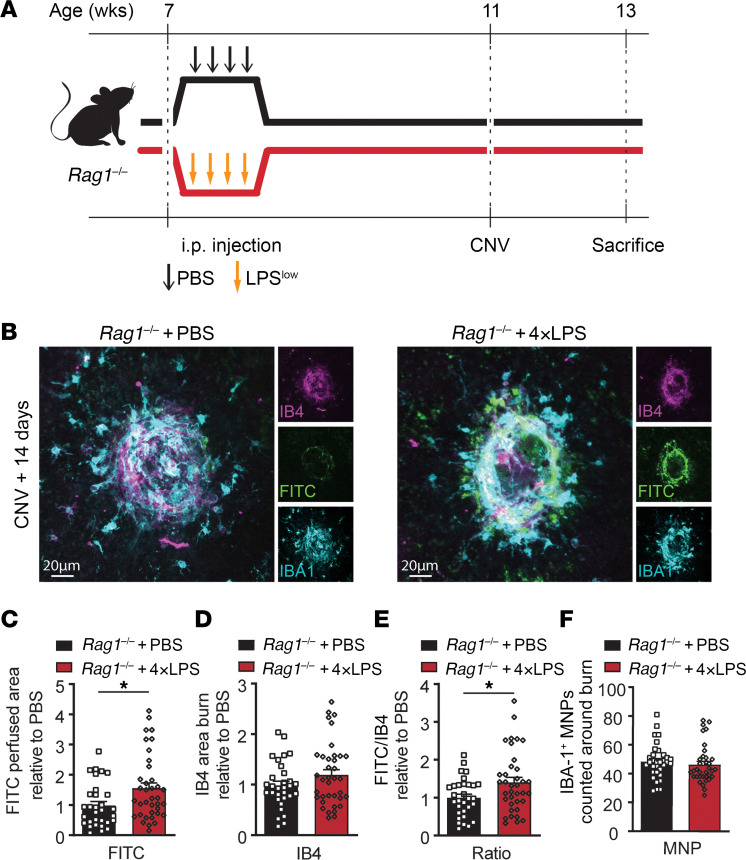
Adaptive immunity is not required for the proangiogenic effect of LPS-induced immune memory. (**A**) Time course of *Rag1^–/–^* mice injected with either 4×LPS or PBS at 7 weeks. Laser-induced CNV occurred at 11 weeks, euthanasia at week 13. (**B**) CNV confocal images of IB4, FITC-dextran, and IBA1 staining from *Rag1^–/–^* + PBS and *Rag1^–/–^* + 4×LPS mice. Scale bars: 20 μm. (**C**–**F**) Quantification of CNV area (**C**), IB4-stained laser impact area (**D**), FITC/IB4 ratio per laser burn (**E**), and number of IBA1-positive MNPs (**F**) on day 14; *n* = 31 burns (PBS), *n* = 37 burns (4×LPS). Data are presented as mean ± SEM. Student’s unpaired *t* test (**C**–**F**) was used. *P < 0.05.

In contrast to innate immunity, the role of adaptive immunity in AMD pathology is limited (36, 37). To determine whether adaptive immunity contributes to the proangiogenic effects of prior exposure to endotoxin, we employed *Rag1^–/–^* mice that lack B and T cells. At 7 weeks of age, these mice were treated with 4×LPS i.p. injections prior to CNV induction at 11 weeks of age (Figure 5A). Preconditioning with 4×LPS increased CNV area in *Rag1^–/–^* mice without affecting absolute numbers of lesion-associated MNPs (Figure 5, B–F), thus further supporting the idea that the proangiogenic effect of LPS-induced immune memory is independent of adaptive immunity. Overall, these data suggest that a past history of peripheral immune stimuli can reprogram local CX3CR1^+^ myeloid cells in distal retinal tissue, leading to persistent changes that suppress the immune response but drive pathological events in the retina.

### Peripheral exposure to endotoxin induces epigenetic reprogramming of retina-resident microglia.

**Figure 6:**
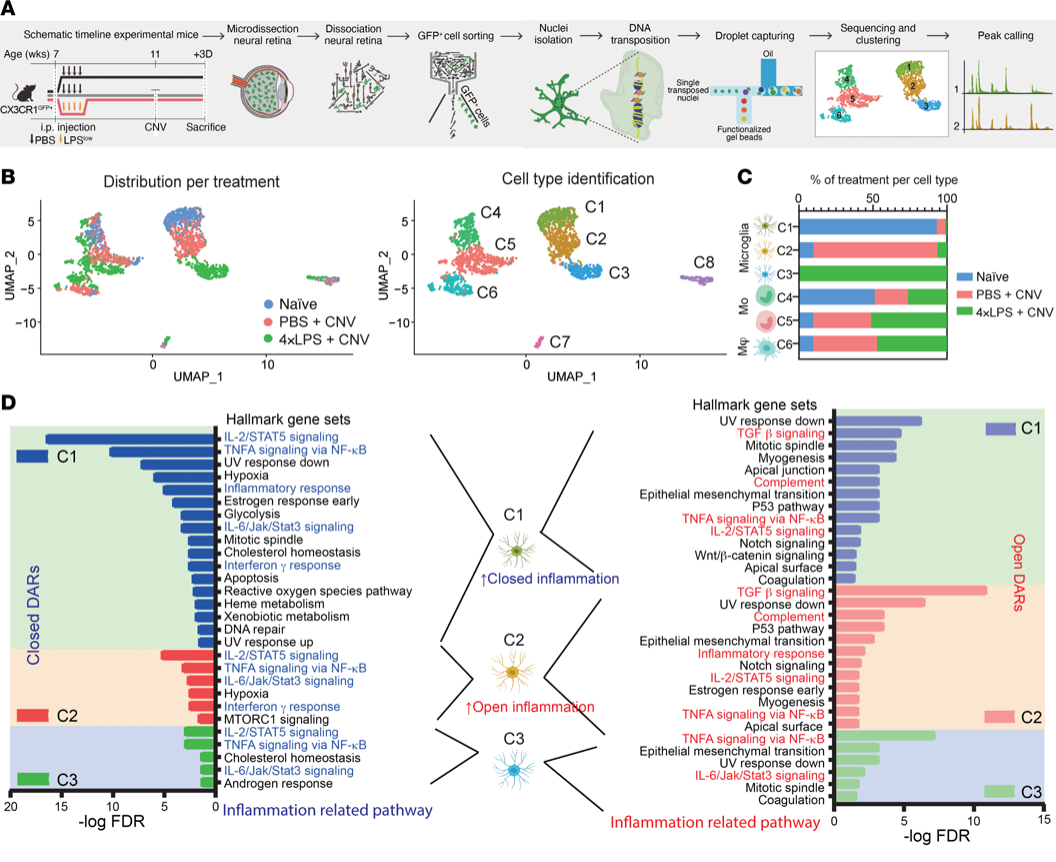
Peripheral exposure to endotoxin induces epigenetic reprogramming of CX3CR1^+^ retina-resident microglia. (**A**) Schematic of the experimental workflow for the single-cell assay for transposase-accessible chromatin sequencing (scATAC-seq) of FACS-isolated CX3CR1^+^ retinal cells from mice 3 days after CNV induction, preconditioned with either PBS or 4×LPS 1 month before, or naive retinas without CNV induction, preconditioned with PBS. (**B**) UMAP projections of scATAC-seq profiles of CX3CR1^+^ retinal myeloid cells (2244 cells from 20 mice) according to sample origin (left) and results of unbiased clustering using the Leiden algorithm (right). Each dot represents an individual cell. (**C**) Bar graphs of sample components of each cluster in **B**. MΦ, macrophage; Mo, monocyte. (**D**) Bar graphs of results of GSEA using differentially accessible regions (DARs) for each microglia cluster enriched in naive, PBS, and 4×LPS groups (C1, C2, and C3, respectively) in closed chromatin regions and open chromatin regions. Inflammation-related pathways are highlighted.

We observed that CX3CR1^+^ myeloid cells in the retina and choroid retain memory and exacerbate neovascularization after peripheral exposure to LPS (Figure 4). CX3CR1^+^ myeloid cells are a heterogeneous population consisting of microglia, circulating monocytes, infiltrating monocytes, and macrophages (38). With the aim of identifying subpopulations of CX3CR1^+^ cells that are epigenetically altered by previous immune stimuli, we performed a single-cell assay for transposase-accessible chromatin with high-throughput sequencing (scATAC-seq) on CX3CR1^+^ retinal myeloid cells extracted from retinas of *Cx3cr1*-EGFP mice. *Cx3cr1*-EGFP mice were engineered with an EGFP sequence inserted into exon 2 of the *Cx3cr1* gene (39). We used heterozygous *Cx3cr1*^GFP/+^ mice since *Cx3cr1*^GFP/GFP^ homozygous mice lack CX3CR1 expression (39). *Cx3cr1*-EGFP mice were subjected to either 4×LPS or PBS only. After 30 days, CNV was induced and EGFP-expressing CX3CR1^+^ cells were sorted. Naive mice injected with PBS and without CNV served as controls (Figure 6A). After quality control, filtering, and dimension reduction, a 2-dimensional uniform manifold approximation and projection (UMAP) was performed on the remaining 835, 821, and 588 cells in the PBS, 4×LPS, and naive groups, respectively. Unbiased clustering was applied using the Leiden algorithm, which partitioned CX3CR1^+^ myeloid cells into 8 distinct clusters (C1–C8). Based on previously described cell-specific gene signatures (40), we identified microglia (clusters C1, C2, and C3), monocytes (clusters C4 and C5), and macrophages (cluster C6) (Figure 6B and Supplemental Figure 4). Gene expression scores identified C4 as circulating monocytes and C5 as a mixture of classical and inflammatory monocytes (40) (Supplemental Figure 5, A–E). Clusters C7 and C8 were photoreceptor and T cell contamination, and were omitted in further analyses. To validate scATAC-seq data, major cluster-specific differentially accessible regions (DARs, defined using adjusted *P* value <0.05 and mean percentage >0.1) were evaluated by chromatin accessibility assay using chromatin from isolated microglia. Gene changes in the promoter regions of hits such as *Tep1* (C1-specific DARs) and *Atf3* (C3-specific DARs) were confirmed, thus attesting to the validity of the approach (Supplemental Figure 5, F and G).

Notably, the 3 microglial populations (C1, C2, and C3) harbored the greatest epigenetic diversity following induction of CNV or preconditioning. Within microglial clusters, distribution varied depending on the group, with the majority of C1 specific to naive control (93.2%), C2 specific to PBS (83.7%), and C3 specific to 4×LPS (99.4%) (Figure 6C). These data suggest that past systemic exposure to LPS modifies CX3CR1^+^ retinal microglia, rendering them epigenetically distinct from microglia in either retinas from naive mice, or from mice undergoing CNV but without prior exposure to LPS. We next performed gene set enrichment analysis (GSEA) using DARs to identify cluster-specific epigenetic changes. GSEA revealed that the C2 subpopulation was characterized by considerable enrichment in open chromatin regions corresponding to genes coding for inflammation-related pathways (TGF-β signaling, complement, inflammatory response, IL-2/STAT5 signaling, and TNF-α signaling via NF-κB) (Figure 6D and Supplemental Figure 5, H and I). In contrast, the C1 subpopulation showed higher numbers of closed chromatin in genes coding for inflammatory processes (Figure 6D). Moreover, when compared with C1 and C2 subpopulations, C3 had open chromatin regions only in 2 gene sets coding for inflammation-related pathways, and 3 gene sets with closed chromatin regions (Figure 6D). Together, these data demonstrate that the chromatin landscape of retina-resident microglia is impacted differently by both prior systemic exposure to LPS and by CNV. CNV itself predisposes retina-resident microglia to a heightened inflammatory response, while prior preconditioning with 4×LPS renders them less prone to an aggressive inflammatory response.

Within monocyte/macrophage clusters, we identified a CNV-associated monocyte cluster (C5) as well as a CNV-associated macrophage cluster (C6) in preconditioned (4×LPS) and control paradigms (PBS) (Figure 6, B and C). Conversely, cluster C4 was not associated with any specific condition. These data suggest that monocytes and macrophages have greater heterogeneity during CNV and that preconditioning with LPS does not epigenetically modify CX3CR1^+^ CNV-associated monocytes/macrophages.

### Peripheral exposure to endotoxin induces transcriptional reprogramming of myeloid cells toward reduced inflammatory but enhanced angiogenic phenotypes.

**Figure 7:**
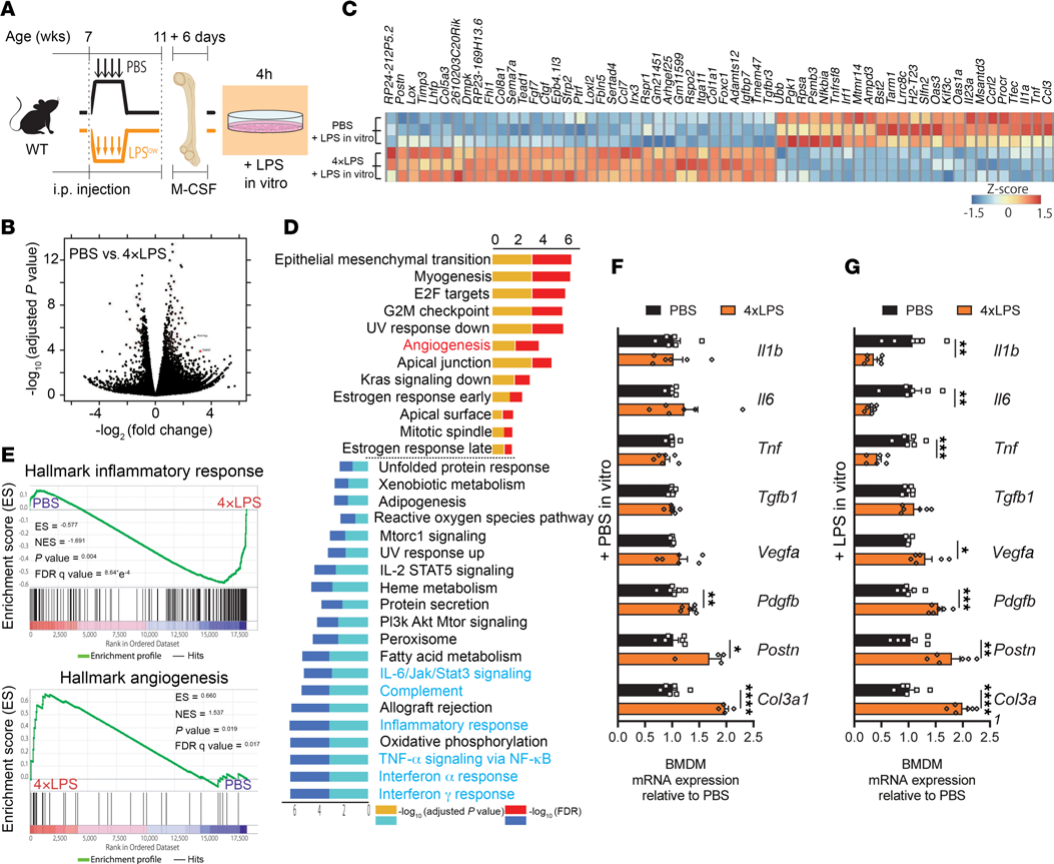
Peripheral exposure to endotoxin induces transcriptional reprogramming of myeloid cells. (**A**) Experimental time course schematic of LPS in vivo and in vitro manipulations (**B**–**G**). C57BL/6J mice were treated with 4×LPS or PBS at 7 weeks of age and BM cells were collected at 11 weeks of age. BM cells were differentiated to BM-derived macrophages (BMDMs) with M-CSF, and BMDMs were harvested after a secondary stimulation with LPS or PBS for 4 hours. Total RNA was extracted and analyzed for gene expression by bulk RNA-seq or qPCR. (**B**) Volcano plot obtained from DESeq2 analysis of LPS-restimulated BMDMs from 4×LPS-prereated mice as compared with LPS-restimulated BMDMs from PBS-pretreated mice. (**C**) Heatmap of the top 60 most differentially expressed genes of LPS-restimulated BMDMs from 4×LPS-pretreated mice as compared with LPS-restimulated BMDMs from PBS-pretreated mice. (**D**) Results of GSEA of Hallmark gene sets showing those enriched in LPS-restimulated BMDMs from mice pretreated with 4×LPS as compared with LPS-restimulated BMDMs from PBS-pretreated mice (FDR < 0.1 and a nominal *P* value < 0.05). A positive normalized enrichment score (NES) value indicates enrichment in the 4×LPS-treated mice. (**E**) GSEA enrichment plots for genes related to inflammatory response and angiogenesis in LPS-restimulated BMDMs from mice pretreated with 4×LPS as compared with LPS-restimulated BMDMs from PBS-pretreated mice; *n* = 3. (**F** and **G**) mRNA expression in BMDMs from PBS-pretreated and 4×LPS-pretreated mice with (**G**) or without LPS restimulation (**F**): *Il1b*, *Il6*, *Tnf*, *Tgfb1*, *Vegfa*, *Pdgfb*, *Postn*, and *Col3a1*; *n* = 6 for all conditions. Data are presented as mean ± SEM. Comparisons between groups were analyzed using Student’s unpaired *t* test (**F** and **G**). *P < 0.05, **P < 0.01, ***P < 0.001, ****P < 0.0001.

To determine whether the distinct chromatin landscapes in microglia and macrophages described above can influence myeloid cell behavior, we next investigated transcriptomic signatures after LPS preconditioning and a secondary challenge. We differentiated bone marrow (BM) cells derived from either control mice or LPS-pretreated mice into mature macrophages (BMDMs), restimulated them with LPS in vitro, and performed bulk RNA sequencing (Figure 7A). The data revealed that pretreatment with LPS, mimicking primary infection sustained over 4 days, leads to significantly altered gene expression (Figure 7, B and C, and Supplemental Figure 6, A–D). Consistent with the global epigenetic silencing of genes coding for inflammation-related processes in retina-resident microglia previously exposed to LPS (Figure 6D), GSEA revealed a negative correlation in mRNA transcripts for processes related to inflammation, such as IFN-γ response, IFN-α response, TNF-α signaling via NF-κB, inflammatory response, complement, and IL-6/Jak/Stat3 signaling, in BMDMs preconditioned with LPS (Figure 7, D and E). Conversely, we observed a positive correlation in clusters of genes coding for angiogenic processes following LPS pretreatment (Figure 7, D and E). RT-qPCR confirmed that proinflammatory genes such as *Il1b*, *Il6*, and *Tnf* decreased significantly in mice preconditioned with LPS, and confirmed an increase in angiogenesis-related genes such as *Vegfa*, *Col3a1*, *Postn*, and *Pdgfb* (Figure 7, F and G). BM had fewer monocytes, especially Ly6C^hi^ inflammatory monocytes, in 4×LPS-pretreated mice compared with PBS-pretreated mice (Supplemental Figure 6, E–H). Collectively, these data suggest that prior exposure to LPS reprograms myeloid cells in the BM as well as retina-resident microglia (but not CNV-associated infiltrating myeloid cells), leading to persistent immune imprinting and enhanced angiogenic responses.

### Prior exposure to peripheral endotoxins shifts myeloid cells toward M2-like polarization.

**Figure 8:**
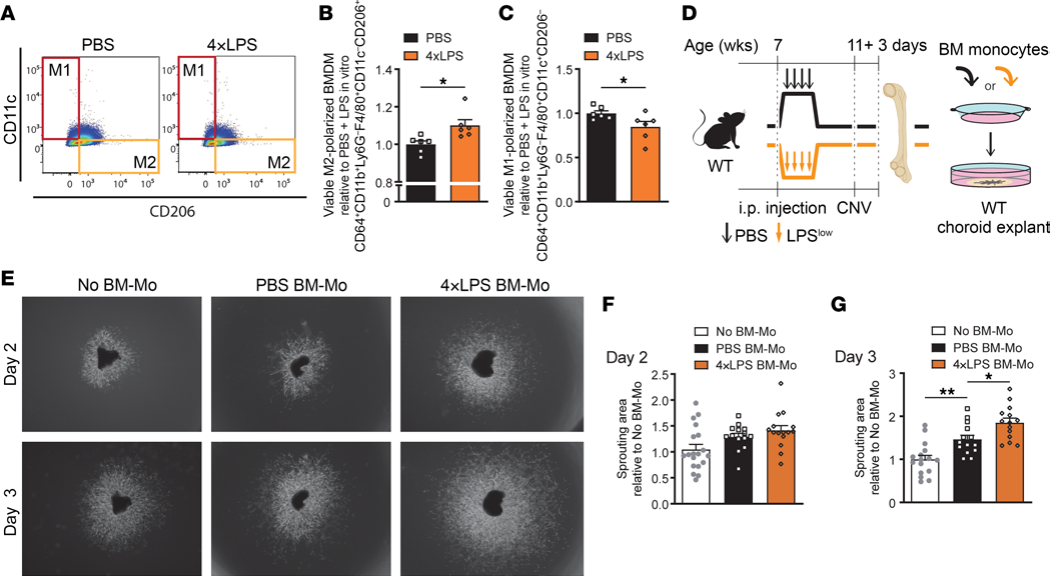
Prior peripheral exposure to endotoxins shifts myeloid cells toward proangiogenic polarization. (**A**) Representative flow cytometry plots of M1- and M2-like macrophages in BMDMs of PBS-pretreated mice and 4×LPS-pretreated mice. (**B** and **C**) Quantification of M2-like macrophages (F4/80^+^CD11b^+^CD11c^−^CD206^+^) (**B**) and M1-like macrophages (F4/80^+^CD11b^+^CD11c^+^CD206^−^) (**C**) in BMDMs of PBS-pretreated mice and 4×LPS-prereated mice (*n* = 6). (**D**) Schematic representation of ex vivo choroid sprouting assay cocultured with BM monocytes (BM-Mo). C57BL/6J mice were treated with 4×LPS or PBS at 7 weeks of age and were subjected to laser burns at 11 weeks. BM cells were collected 3 days after laser burn and BM-Mo were isolated using immunomagnetic negative selection. Choroid pieces (RPE-choroid-sclera from peripheral retina of 5-week-old C57BL/6J mice) were seeded into 24-well plates containing Matrigel and cocultured with BM-Mo using Transwell inserts. (**E**) Representative images of choroid explants at 2 and 3 days of coculture with BM-Mo from PBS-pretreated and 4×LPS-pretreated mice, and without BM-Mo. (**F** and **G**) Quantitation of sprouting area at 2 (**F**) and 3 days (**G**) of coculture with BM-Mo from each group when compared with no BM-Mo. At 2 days, *n* = 19 (No BM-Mo), 14 (PBS BM-Mo), *n* = 15 (4×LPS BM-Mo). At 3 days, *n* = 16 (No BM-Mo), 14 (PBS BM-Mo), *n* = 14 (4×LPS BM-Mo). Data are presented as mean ± SEM. Comparisons between groups were analyzed using Student’s unpaired *t* test (**B** and **C**) or 1-way ANOVA with Tukey’s multiple-comparison test (**F** and **G**). *P < 0.05; **P < 0.01.

Myeloid cells, including microglia and monocytes/macrophages, polarize to classically activated/proinflammatory (M1-like) or alternatively activated/antiinflammatory and proreparative (M2-like) phenotypes (41, 42). We assessed polarization of BMDMs from LPS-preconditioned mice by flow cytometry and found a significant increase in CD206-expressing M2-like cells (Figure 8, A and B) and a decrease in M1-like cells (Figure 8, A and C). Rebalancing of myeloid cells toward a M2-like state can contribute to disorders linked to heightened angiogenesis (43); therefore, prior exposure to LPS could exacerbate CNV.

To determine the impact on microvascular angiogenesis by myeloid cells previously exposed to peripheral endotoxin, we isolated monocytes from the BM of laser-burned mice injected with PBS or preconditioned with 4×LPS. Monocytes were then cocultured with choroidal explants, and angiogenesis was evaluated 2 and 3 days later (Figure 8D). We observed a significant increase in sprouting area in choroid explants cocultured with myeloid cells from 4×LPS-primed mice when compared with PBS-primed control mice (Figure 8, E-G). Taken together, these data support the notion that prior exposure to endotoxins such as LPS renders myeloid cells less proinflammatory and more proangiogenic and is consistent with increased CNV (Figure 2) and reprogrammed chromatin landscapes (Figures 3–7) observed in LPS-primed mice. These findings are consistent with other studies suggesting that compromised inflammatory responses may aggravate the laser-induced CNV lesions in mice (44, 45).

### Peripheral exposure to bacterial endotoxin suppresses inflammatory and potentiates angiogenic responses in myeloid cells via ATF3 deregulation.

**Figure 9:**
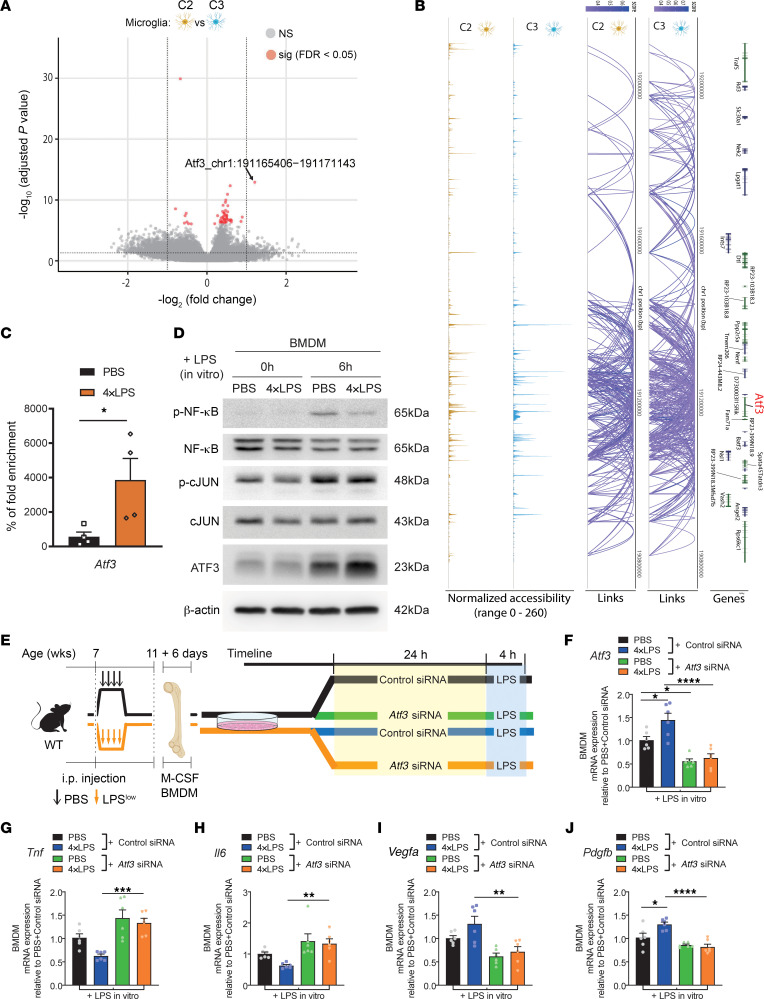
Peripheral exposure to endotoxins modulates myeloid cell response via ATF3 deregulation. (**A**) Volcano plot of accessible regions with differentially accessible regions (DARs; defined by an FDR adjusted *P* value < 0.05, total of 51 DARs) identified between comparisons of microglial 4×LPS cluster (C3) versus microglial PBS cluster (C2) as found by CX3CR1^+^ retinal myeloid cell scATAC-seq data. (**B**) *Cis*-coaccessibility network (CCAN) links between the *ATF3* promoter and distal sites in the surrounding region generated by subset analysis by cluster. Connections from microglial PBS cluster (C2) and microglial 4×LPS cluster (C3) are shown, with a minimum coaccessibility score of 0.3. (**C**) Chromatin accessibility in the promoter region of *Atf3* in BMDMs from PBS-pretreated and 4×LPS-pretreated mice as analyzed by qPCR. *n* = 4 (PBS), *n* = 4 (4×LPS). (**D**) Representative immunoblots showing p-NF-κB, total NF-κB, p-c-JUN, total c-JUN, and ATF3 expression in BMDMs from PBS-pretreated and 4×LPS-pretreated mice with and without LPS restimulation. (**E**) Schematic representation of knockdown of *Atf3* gene expression in BMDMs using siRNA. C57BL/6J mice were treated with 4×LPS or PBS at 7 weeks of age and BM cells were collected at 11 weeks of age. BM cells were differentiated into BMDMs with M-CSF. BMDMs were transfected with *Atf3* or control siRNA for 24 hours and then restimulated with LPS for 4 hours. RNA was extracted for qPCR analysis. (**F**–**J**) mRNA expression in BMDMs transfected with *Atf3* or control siRNA from PBS-pretreated and 4×LPS-pretreated mice with LPS restimulation: *Atf3* (**F**), *Tnf* (**G**), *Il6* (**H**), *Vegfa* (**I**), and *Pdgfb* (**J**); *n* = 6 for all groups. Data are presented as mean ± SEM. Comparisons between groups were analyzed using Student’s unpaired *t* test (**C**) or 1-way ANOVA with Tukey’s multiple-comparison test (**F**–**J**). *P < 0.05; **P < 0.01; ***P < 0.001; ****P < 0.0001.

In order to gain insight into the mechanism by which exposure to endotoxin reprograms microglial cells directly in the retina, we analyzed scATAC-seq data on nuclei extracted from CX3CR1^+^ cells enriched from retinas (Figure 6). We identified DARs for activating transcription factor 3 (ATF3) as the most enriched accessible region when comparing microglia from the PBS cluster C2 (primarily microglia from PBS + CNV) and microglial 4×LPS cluster C3 (primarily microglia from 4×LPS + CNV) (Figure 9A). We then investigated *cis*-coaccessibility networks (CCANs), which are modules of sites that are highly coaccessible with one another, using Cicero (46). We found that CCANs that include ATF3 have greater accessibility signals in C3 (4×LPS) microglia when compared with control C2 (PBS) microglia (Figure 9B), suggesting that the *Atf3* gene is preferentially epigenetically modulated following exposure to endotoxin, and, hence, could potentially be a regulator of LPS-driven immune memory.

ATF3 is a member of the ATF/cAMP response element–binding (CREB) family, and it is a stress-induced transcription factor that plays pivotal roles in modulating glucose metabolism and immune responses (47). ATF3 can act as either a transcription activator or repressor. We examined the functional role of ATF3 in LPS-driven innate immune memory using BMDMs from mice preconditioned by 4×LPS or PBS control. As with C3 microglia, enhanced chromatin accessibility in the promoter region of *Atf3* was observed in BMDMs from 4×LPS-pretreated mice (Figure 9C). Pretreatment of mice with PBS and restimulation with LPS for 6 hours led to a robust induction of ATF3 as well as p-cJun and p-NF-κB, 2 phosphorylated transcription factors involved in TLR4 signaling (48) (Figure 9D). Conversely, in agreement with previous reports suggesting that ATF3 negatively regulates TLR4 signaling (49), we found that secondary exposure to LPS upon prior priming with 4×LPS further induced ATF3, but attenuated induction of p-NF-κB (Figure 9D).

Finally, we examined the effect of ATF3 knockdown on myeloid cell memory (Figure 9E). BMDMs from mice exposed to 4×LPS or PBS control were transfected with *Atf3* siRNAs for 24 hours and then treated with LPS for 4 hours. We confirmed efficacy of siRNAs against *Atf3* in both preconditioning paradigms (PBS or 4×LPS) by qPCR (Figure 9F). Consistent with our previous findings (Figure 3), genes coding for inflammatory response such as *Tnf* and *Il6* were downregulated following secondary exposure to LPS in 4×LPS-primed BMDMs treated with control siRNA (Figure 9, G and H). When *Atf3* was silenced, both *Tnf* and *Il6* were significantly upregulated in the 4×LPS-primed group to levels observed in control PBS-injected groups (Figure 9, G and H). These data suggest that ATF3 and its induction contributes to mitigating inflammatory phenotypes in endotoxin-driven immune memory. Further, the induction of proangiogenic genes *Vegfa* and *Pdgfb* in 4×LPS-primed groups treated with control siRNA was significantly blunted when *Atf3* was silenced (Figure 9, I and J). Collectively, these data identify ATF3 as a mediator of innate immune memory following endotoxin exposure and implicate it in regulating inflammatory and angiogenic responses in retina-resident myeloid cells.

## Discussion

The innate immune system has evolved as the first line of defense against pathogens and is sufficient for plants, invertebrates, and against most challenges in vertebrates (50, 51). The idea that the primitive cells that comprise the innate immune system, such as MNPs, have memory has been taking shape over the past decade and challenging a long-held dogma that only adaptive immunity holds potential for remembering prior stimuli (52, 53). In the current study, we outline a mechanism by which prior immunological events can reprogram myeloid cells such as retina-resident microglia and BMDMs. We demonstrate their impact on aggravating pathological angiogenesis in a model of nvAMD and show that short-lived immunostimulatory events, such as infection with *C*. *pneumoniae* or peripheral exposure to low doses of endotoxin, lead to long-term epigenetic modifications of CX3CR1^+^ retina-resident MNPs. This renders CX3CR1^+^ MNPs susceptible to trigger more robust proangiogenic responses upon subsequent exposure to stimuli while blunting inflammatory cytokine production. The epigenetic imprinting lasts long after the initial inflammation has resolved. It may be a vestigial response where a primary exposure to a pathogen is met with a destructive immune response and, subsequently, the same sentinel cells orchestrate a reparative process to reinstate homeostasis. Yet, in the retina, this is detrimental, as reparative MNPs secrete proangiogenic growth factors that partake in pathological angiogenesis.

Diseases of the aging CNS, such as AMD, may be of particular susceptibility to imprinting from past immunological events. Given that these events occur over an entire span of life, long-lived retina-resident myeloid cells such as microglia are likely candidates to be affected by this phenomenon (54, 55). Cells of myeloid lineage, in particular monocytes and their progeny, have a propensity to retain long-term functional memory following exposure to infectious agents and vaccinations (50, 51). Exposure to fungally derived polysaccharide β-glucan or the bacille Calmette-Guerin (BCG) vaccine can promote a sustained enhanced response of myeloid cells to secondary infectious or inflammatory challenges. This process has been termed ‘‘trained immunity’’ (56, 57). Conversely, our data suggest that prior exposure to endotoxin or LPS, a major component of the outer membrane of Gram-negative bacteria, can condition MNPs to hinder the inflammatory response when a second stimulation occurs. These findings are consistent with previous data and broadly termed ‘‘immune tolerance’’ (29, 31, 33).

Innate immune memory is mediated via epigenetic, transcriptomic, and metabolic reprogramming. Using scATAC-seq and targeted elimination of a specific immune cell population, we demonstrated that peripheral low-dose exposure to LPS reprograms tissue-resident microglia within the distal tissue of the retina, which is protected by the BRB. These findings agree with a recent report showing LPS-driven innate immune memory in tissue-resident microglia in the brain (29).

To date, monocytes and macrophages are the best-characterized effectors of innate immune memory (30, 32, 50, 57). While mature myeloid cells such as monocytes are short-lived, with an average half-life of 5 to 7 days (58), β-glucan and BCG train long-lived myeloid progenitor cells in the BM and lead to lasting production of trained circulating effector cells (59–61). In contrast to monocytes or monocyte-derived cells, tissue-resident microglia in the CNS (brain and retina), which arise from yolk sac precursor cells during early embryogenesis, are very long-lived, maintaining a self-renewal ability even into adulthood (62). This enables tissue-resident microglia to retain the long-term history of immune challenge and modulate their response accordingly. Heterogeneity in microglial populations is well established, as distinct microglia subtypes exhibit divergent enhancer landscapes, gene expression profiles, morphologies, or functions (63–65). Remarkably, our scATAC-seq analysis shows that almost all retina-resident microglia are epigenetically reprogrammed by peripheral low-dose LPS. This suggests immunological memory may be a fundamental property in microglia and their behavior could be easily and remotely altered by immunological events. scATAC-seq analysis also identified ATF3 as the most accessible chromatin region in retina-resident myeloid cells that have been previously exposed to peripheral LPS. Increased ATF3 expression was also noted in response to secondary stimulation with LPS. ATF3 stands as a key transcription regulator that inhibits the inflammatory response and also regulates M2 polarization through the Wnt/β‑catenin signaling pathway (66). ATF3 knockdown confirmed its essential role in regulating the shift of myeloid cells toward a proangiogenic and less inflammatory state in LPS-driven memory.

An association between *C*. *pneumoniae* and exacerbation of AMD has been documented in multiple human studies (21, 22, 67); however, a direct mechanistic cause-and-effect relationship had yet to be conclusively elucidated. *C*. *pneumoniae* is a common cause of community-acquired pneumonia. Over 50% of the adult population carry anti–*C*. *pneumoniae* antibodies (68), but not all patients infected with *C*. *pneumoniae* progress to AMD. This may be explained by the fact that most individuals have subclinical infection with or without mild symptom of pneumonia and our data suggest that short exposure to endotoxin only leads to weak proangiogenic profiles. A parallel may be drawn with recent evidence suggesting that the risk of developing multiple sclerosis (MS) increased 32-fold following Epstein-Barr virus (EBV) infection (69). However, 95% of adults have prior EBV infection and yet well under 0.25% develop MS. Hence, additional genetic and environmental factors may modulate or trigger disease.

In summary, short-term peripheral inflammation has the propensity to induce long-term chromatin remodeling in tissue-resident myeloid cells within the CNS, such as microglia. Ultimately, this can influence distal pathological angiogenesis in diseases such as AMD. Given that retinal microglia are long-lived, these findings provide insight into how immunological events incurred over a lifetime can influence and perhaps initiate the course of neuroinflammatory diseases within the eye. More broadly, our work provides a framework for a better understanding of the long-term risk of AMD progression in patients with a past history of infection or systemic inflammatory diseases. Future therapeutic avenues to influence epigenetic reprogramming of the innate immune system may have rationale to delay or prevent the onset of AMD.

## Methods

Detailed methods can be found in the supplemental material.

### Mice.

Animals were housed in the animal facility of the Hospital Maisonneuve-Rosemont Research Center under a 12-hour light/dark cycle with ad libitum access to food and water unless indicated otherwise. Only male mice were used in this study and were enrolled in the different studies at 6 or 7 weeks of age.

C57BL/6J and homozygous B6.129S7-*Rag1^tm1Mom^*/J (referred to as *Rag1^–/–^*) mice were purchased from The Jackson Laboratory and bred at the Hospital Maisonneuve-Rosemont Research Center animal facility.

Homozygous B6.129P2(C)-*Cx3cr1^tm2.1(cre/ERT2)Jung^*/J (referred to as *Cx3cr1*^CreER^) mice were crossed in-house with homozygous C57BL/6-*Gt(ROSA)26Sor^tm1(HBEGF)Awai^*/J (referred to as R26^iDTR^) mice to obtain heterozygous *Cx3cr1*^CreER/+^:R26^iDTR/+^ mice. Homozygous B6.129P-*Cx3cr1^tm1Litt^*/J (referred to as *Cx3cr1*^GFP^ mice) were crossed in-house with C57BL/6J mice to obtain heterozygous *Cx3cr1*^GFP/+^ mice.

### Data and code availability.

scATAC-seq data for the study are deposited in NCBI’s Gene Expression Omnibus and are accessible through GEO accession no. GSE194226.

This study did not generate new unique code.

### Statistics.

Data are expressed as mean ± SEM, unless indicated otherwise. Multiple Student’s *t* tests, 2-tailed Student’s *t* test, or ANOVA *t* test were used to compare 2 or more than 2 groups, respectively. Statistical significance was considered when *P* was less than 0.05 and is noted as *P < 0.05, **P < 0.01, ***P < 0.001, and ****P < 0.0001. All experimental *n* values are indicated in the figure legends.

### Study approval.

All animal studies were performed in compliance with the ARRIVE guidelines and the Association for Research in Vision and Ophthalmology (ARVO) Statement for the Use of Animals in Ophthalmic and Vision Research and were approved by the Animal Care Committee of the Maisonneuve-Rosemont Hospital Research Center in agreement with the guidelines established by the Canadian Council on Animal Care.

## Author contributions

Masayuki H and PS designed research. Masayuki H, Maki H, EMMAA, RDM, RJ, FP, VG, FF, MB, EH, and AMW performed research. Masayuki H, Maki H, EMMAA, CG, VC, EH, and PS analyzed data. Masayuki H, Maki H, EMMAA, SCG, RDM, FB, FF, CG, VC, HJM, JSJ, AMW, and PS wrote the manuscript and made figures. All authors revised and agreed on the final version of the manuscript.

## Supplementary Material

Supplemental data

## Version 1 02/15/2023

Electronic publication
