# Supplementary material for: Early-life peripheral infections reprogram retinal microglia and aggravate neovascular age-related macular degeneration in later life
Source: J Clin Invest. 2023 Feb 15;133(4):e159757. doi: 10.1172/JCI159757 (PMC9927938; doi:10.1172/JCI159757)
Supplement: Supplemental data [file jci-133-159757-s087.pdf]

Figure S1

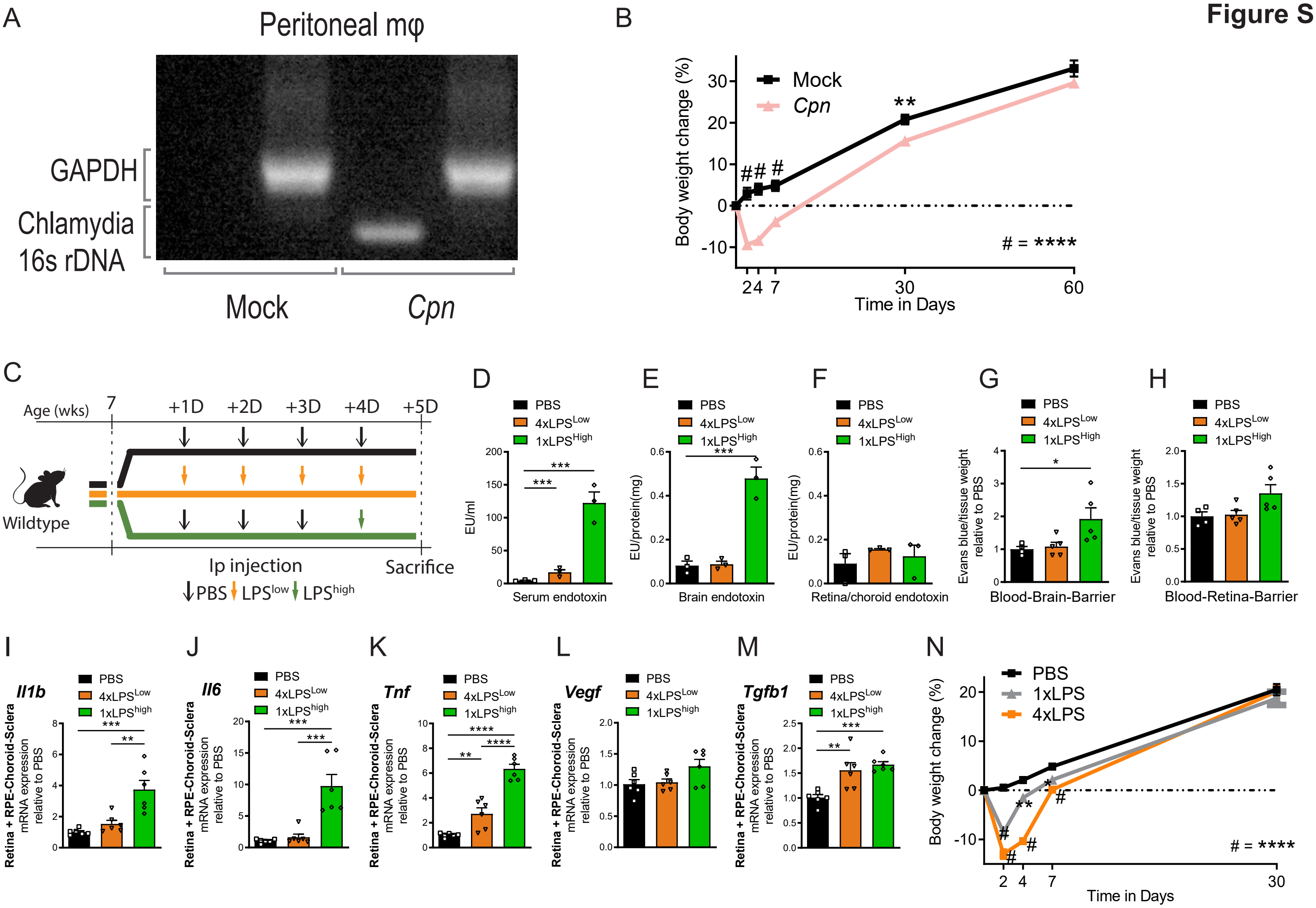
A
Peritoneal mφ
GAPDH
Chlamydia 16s rDNA
Mock
Cpn
B
Body weight change (%)
Mock
Cpn
Time in Days
# = ****
C
Age (wks)
+1D
+2D
+3D
+4D
+5D
Wildtype
Ip injection
Sacrifice
PBS LPSlow LPShigh
D
Serum endotoxin
E
Brain endotoxin
F
Retina/choroid endotoxin
G
Blood-Brain-Barrier
H
Blood-Retina-Barrier
I
Il1b
J
Il6
K
Tnf
L
Vegf
M
Tgfb1
Retina + RPE-Choroid-Sclera mRNA expression relative to PBS
N
PBS
1xLPS
4xLPS
Time in Days

Figure S2

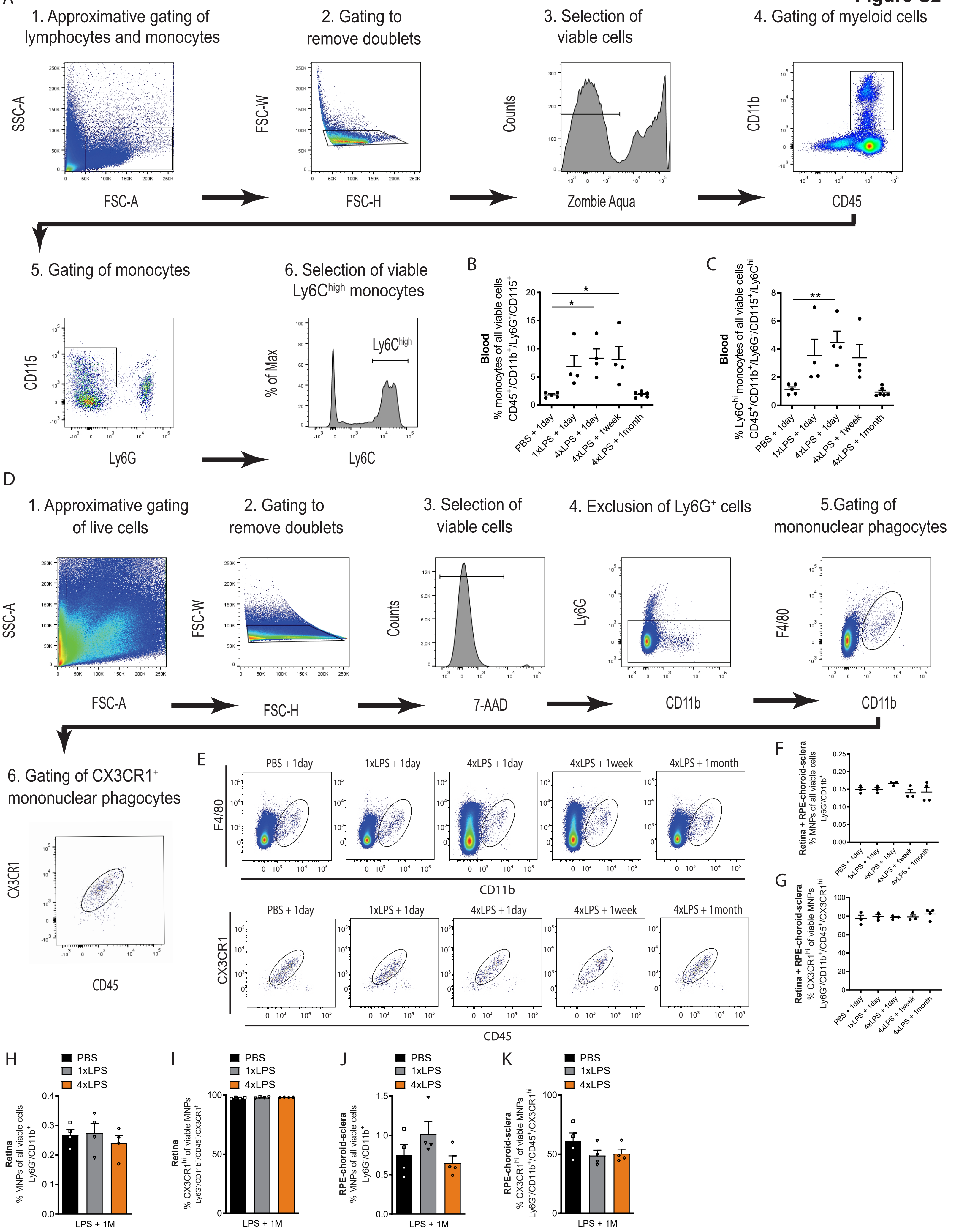

A
1. Approximative gating of lymphocytes and monocytes
2. Gating to remove doublets
3. Selection of viable cells
4. Gating of myeloid cells
SSC-A
FSC-A
FSC-W
FSC-H
Counts
Zombie Aqua
CD11b
CD45
5. Gating of monocytes
6. Selection of viable Ly6Chigh monocytes
CD115
Ly6G
% of Max
Ly6C
Ly6Chigh
B
Blood
% monocytes of all viable cells CD45+/CD11b+/Ly6G-/CD115+
PBS + 1day
1xLPS + 1day
4xLPS + 1day
4xLPS + 1week
4xLPS + 1month
C
Blood
% Ly6Chi monocytes of all viable cells CD45+/CD11b+/Ly6G-/CD115+/Ly6Chi
D
1. Approximative gating of live cells
2. Gating to remove doublets
3. Selection of viable cells
4. Exclusion of Ly6G+ cells
5.Gating of mononuclear phagocytes
7-AAD
Ly6G
F4/80
6. Gating of CX3CR1+ mononuclear phagocytes
CX3CR1
E
F
Retina + RPE-choroid-sclera
% MNPs of all viable cells Ly6G-/CD11b+
G
Retina + RPE-choroid-sclera
% CX3CR1hi of viable MNPs Ly6G-/CD11b+/CD45+/CX3CR1hi
H
PBS
1xLPS
4xLPS
Retina
% MNPs of all viable cells Ly6G-/CD11b+
LPS + 1M
I
Retina
% CX3CR1hi of viable MNPs Ly6G-/CD11b+/CD45+/CX3CR1hi
J
RPE-choroid-sclera
% MNPs of all viable cells Ly6G-/CD11b+
K
RPE-choroid-sclera
% CX3CR1hi of viable MNPs Ly6G-/CD11b+/CD45+/CX3CR1hi

Figure S3

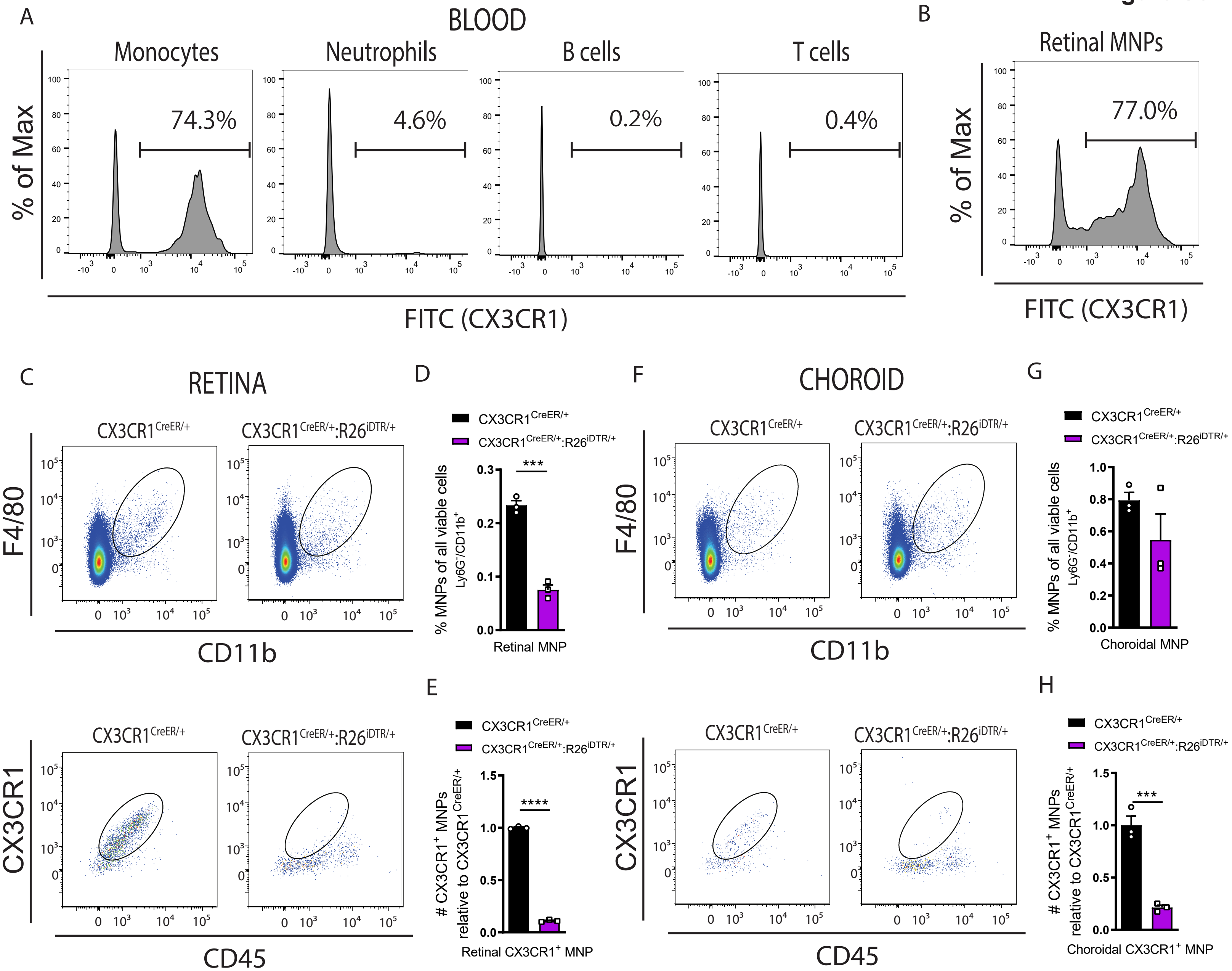
A
BLOOD
Monocytes
Neutrophils
B cells
T cells
% of Max
74.3%
4.6%
0.2%
0.4%
FITC (CX3CR1)
B
Retinal MNPs
77.0%
% of Max
FITC (CX3CR1)
C
RETINA
CX3CR1CreER/+
CX3CR1CreER/+:R26iDTR/+
F4/80
CD11b
CX3CR1
CD45
D
% MNPs of all viable cells Ly6G-/CD11b+
Retinal MNP
***
E
CX3CR1+ MNPs relative to CX3CR1CreER/+
Retinal CX3CR1+ MNP
****
F
CHOROID
G
Choroidal MNP
H
Choroidal CX3CR1+ MNP
***

Figure S4

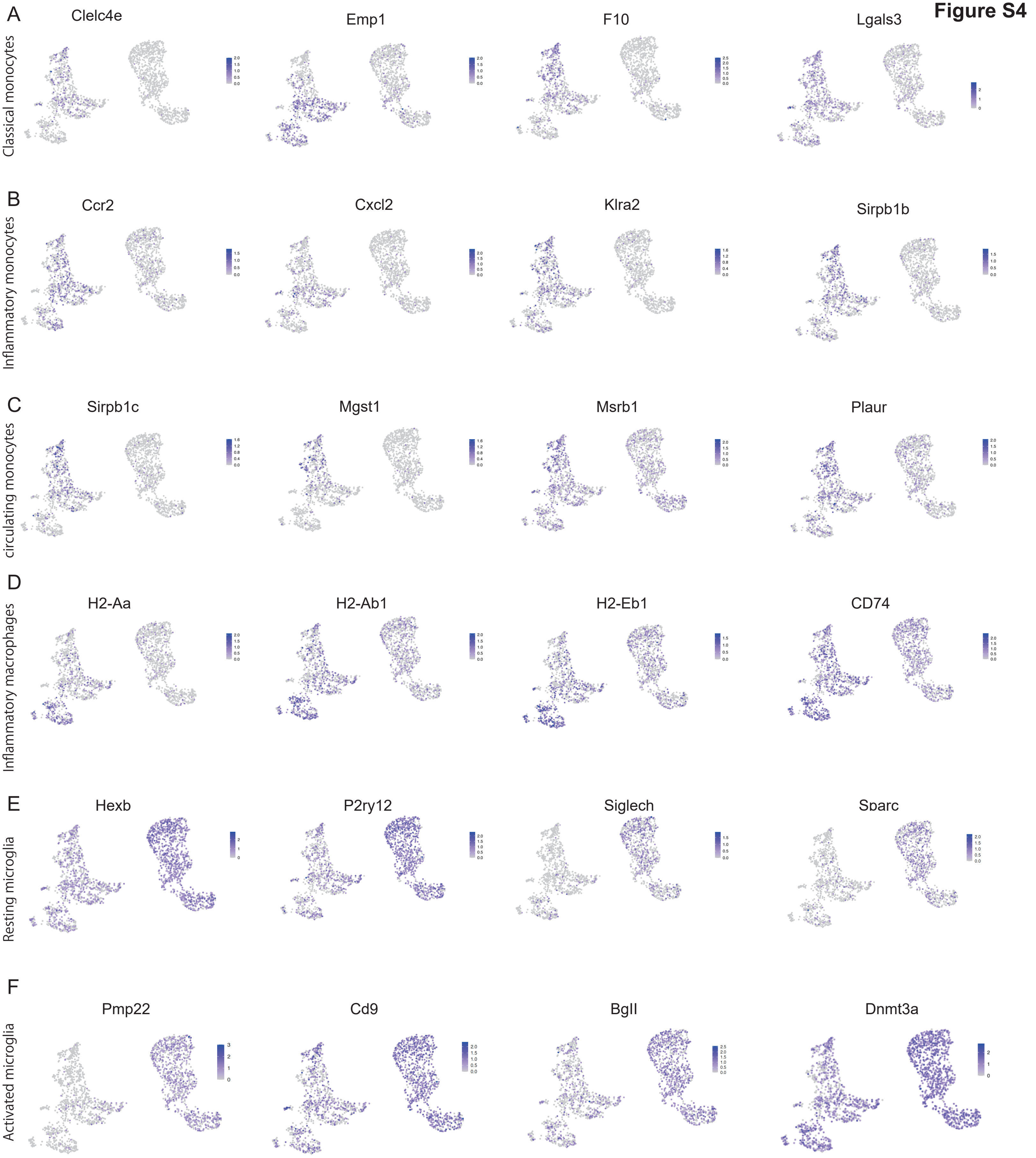
A
Classical monocytes
Clelc4e
Emp1
F10
Lgals3
B
Inflammatory monocytes
Ccr2
Cxcl2
Klra2
Sirpb1b
C
circulating monocytes
Sirpb1c
Mgst1
Msrb1
Plaur
D
Inflammatory macrophages
H2-Aa
H2-Ab1
H2-Eb1
CD74
E
Resting microglia
Hexb
P2ry12
Siglech
Sparc
F
Activated microglia
Pmp22
Cd9
BglI
Dnmt3a

Figure S5

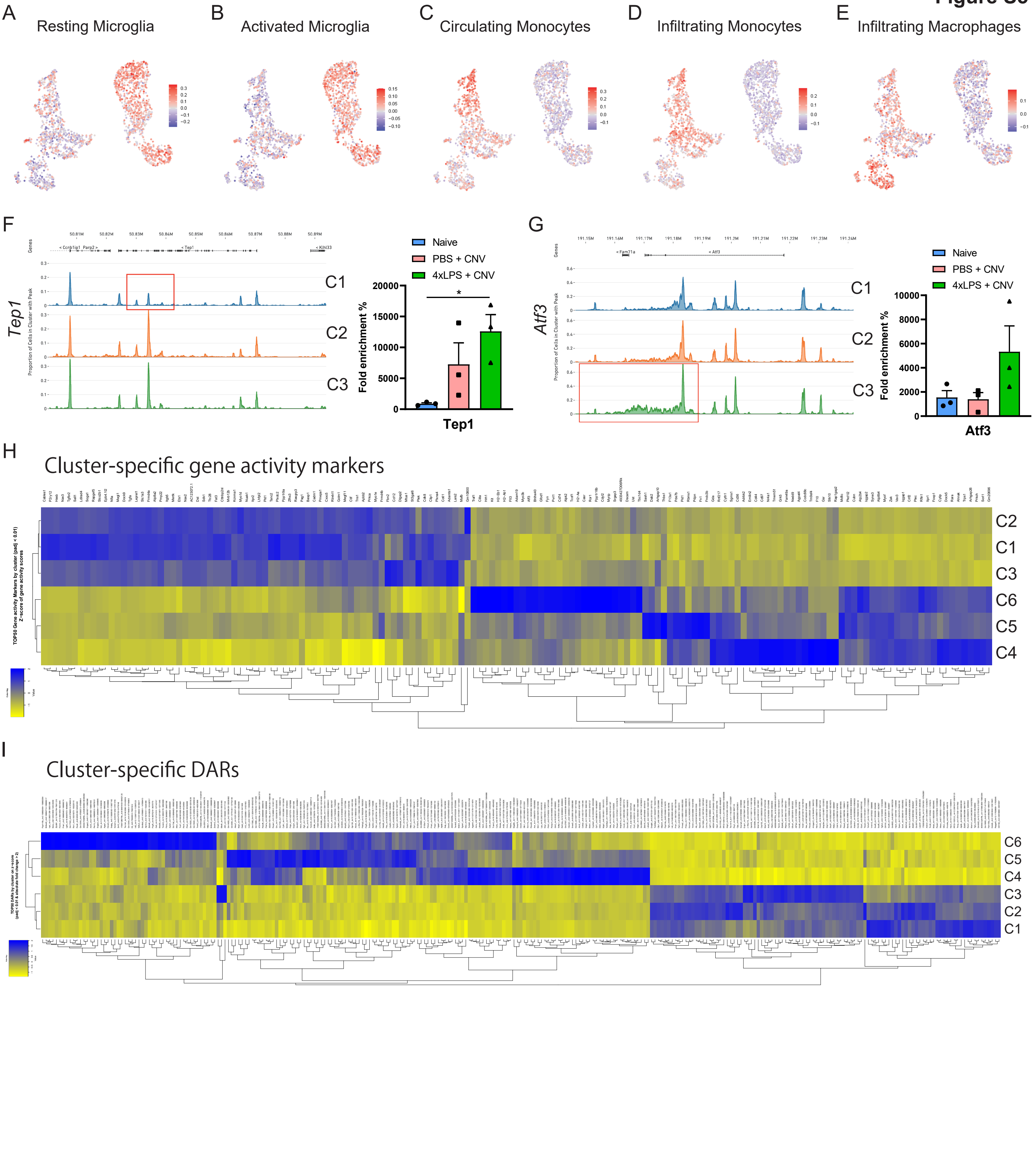

A
Resting Microglia
B
Activated Microglia
C
Circulating Monocytes
D
Infiltrating Monocytes
E
Infiltrating Macrophages
F
Tep1
C1
C2
C3
Proportion of Cells in Cluster with Peak
Naive
PBS + CNV
4xLPS + CNV
Fold enrichment %
Tep1
G
Atf3
C1
C2
C3
Proportion of Cells in Cluster with Peak
Naive
PBS + CNV
4xLPS + CNV
Fold enrichment %
Atf3
H
Cluster-specific gene activity markers
C2
C1
C3
C6
C5
C4
I
Cluster-specific DARs
C6
C5
C4
C3
C2
C1

Figure S6

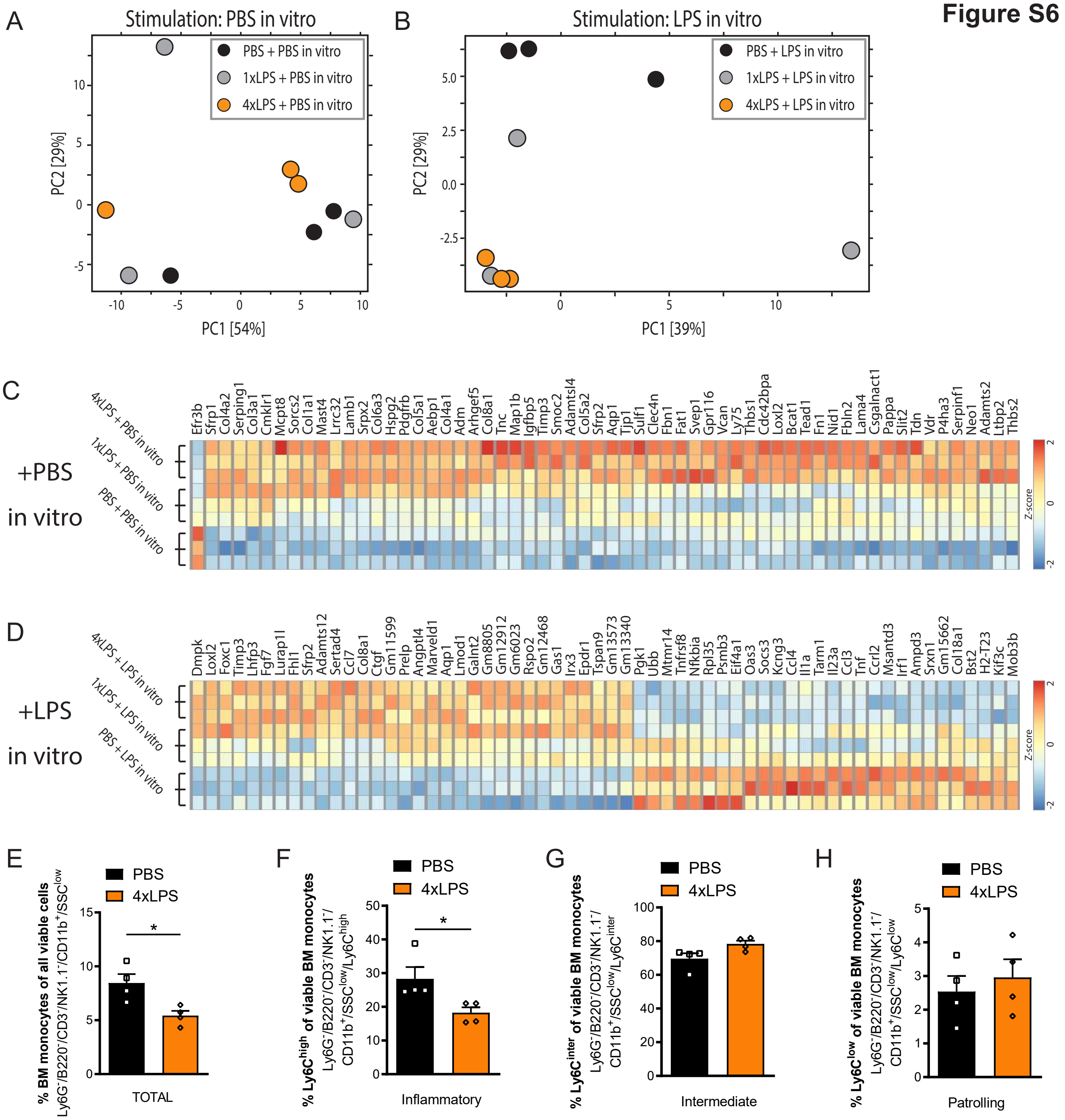
A
Stimulation: PBS in vitro
PBS + PBS in vitro
1xLPS + PBS in vitro
4xLPS + PBS in vitro
PC1 [54%]
PC2 [29%]
B
Stimulation: LPS in vitro
PBS + LPS in vitro
1xLPS + LPS in vitro
4xLPS + LPS in vitro
PC1 [39%]
PC2 [29%]
C
+PBS in vitro
4xLPS + PBS in vitro
1xLPS + PBS in vitro
PBS + PBS in vitro
Z-score
D
+LPS in vitro
4xLPS + LPS in vitro
1xLPS + LPS in vitro
PBS + LPS in vitro
Z-score
E
PBS
4xLPS
% BM monocytes of all viable cells
TOTAL
F
PBS
4xLPS
Inflammatory
G
PBS
4xLPS
Intermediate
H
PBS
4xLPS
Patrolling

SUPPLEMENTAL FIGURE LEGENDS

Supplemental Figure 1: Effect of *in vivo* exposure to *Chlamydia pneumoniae* (*Cpn*) or LPS.

(A) Agarose gel showing PCR amplification results confirming the existence of *Cpn* ribosomal DNA in peritoneal macrophages from infected mice at 3 days post infection.

(B) Percentage weight gain of mice previously infected with *Cpn* (n = 9) and mock-infected (n = 7).

(C) Schematic representation of experimental timecourse where mice were injected with either a single intraperitoneal injection of LPS at a high-dose of 5 mg/kg, following vehicle (PBS) injections for three days (high-dose LPS; $1xLPS^{high}$), daily intraperitoneal injections of LPS at a low-dose of 0.5 mg/kg on four consecutive days (low-dose LPS; $4xLPS^{low}$), or daily intraperitoneal injections of vehicle (PBS).

(D-F) Endotoxin levels in blood **(D)**, brain **(E)**, and retina/choroid **(F)** 24 hours after the last injection; n= 3 (PBS), n= 3 ($4xLPS^{low}$), n= 3 ($1xLPS^{high}$).

(G, H) Evans blue was extracted and quantified from the brain surface **(G)** and retina **(H)** 24 h after the last injection, n= 4 (PBS), n= 5 ($4xLPS^{low}$), n= 5 ($1xLPS^{high}$).

(I-M) mRNA expression in retina/RPE-choroid-sclera complexes 4 weeks 24 hours after the last injection relative to PBS of *Il1b* (**I**), n= 6 (PBS), n= 6 ($4xLPS^{low}$), n= 6 ($1xLPS^{high}$); *Il6* (**J**), n= 6 (PBS), n= 6 ($4xLPS^{low}$), n= 6 ($1xLPS^{high}$); *Tnf* (**K**), n= 6 (PBS), n= 6 ($4xLPS^{low}$), n= 6 ($1xLPS^{high}$); *Vegf* (**L**), n= 6 (PBS), n= 6 ($4xLPS^{low}$), n= 6 ($1xLPS^{high}$); *Tgfb1* (**M**), n= 6 (PBS), n= 6 ($4xLPS^{low}$), n= 6 ($1xLPS^{high}$).

(N) Percentage weight gain of mice; n= 17 (PBS), n= 18 (1xLPS), n= 17 (4xLPS).

Comparisons between groups were analyzed using one-way ANOVA with Tukey's multiple comparisons test (**D-M**), and two-way ANOVA with Sidak's multiple comparisons test (**B, N**); *P < 0.05, **P < 0.01, ***P < 0.001, ****P< 0.0001; error bars represent mean ± SEM.

Supplemental Figure 2: Timecourse of changes of circulating and retinal myeloid cells after LPS injections.

(A) Gating scheme for blood monocytes: 1) approximative gating of lymphocytes and monocytes, 2) gating to remove doublets, 3) selection of viable cells, 4) gating of myeloid cells, 5) gating of viable monocytes, and 6) selection of viable $Ly6C^{high}$ monocytes.

(B, C) Flow cytometry analysis of blood from mice 24 hours after PBS injection, 1xLPS injection, and 4xLPS injections, 1 week after 4xLPS injections, and 4 weeks after

4xLPS injections. Quantification of the percentage of viable monocytes ($CD45^+/CD11b^+/Ly6G^-/CD115^+$) (**B,** n=5, 4, 4, 4, 6 mice from left to right) and viable $Ly6C^{high}$ monocytes ($CD45^+/CD11b^+/Ly6G^-/CD115^+/Ly6C^{high}$) (**C,** n=5, 4, 4, 4, 6 mice from left to right) of total leukocytes.

(D) Gating scheme for mononuclear phagocytes (MNPs) in whole retinas and RPE-choroid-sclera complexes: 1) approximative gating of live cells, 2) gating to remove doublets, 3) selection of viable cells, 4) exclusion of Ly6G positive cells, 5) gating of MNPs, and 6) gating of $CX3CR1^+$ MNPs.

(E-G) Flow cytometry analysis of whole retinas and RPE-choroid-sclera complexes from mice 24 hours after PBS injection, 1xLPS injection, and 4xLPS injections, 1 week after 4xLPS injections, and 4 weeks after 4xLPS injections. Representative FACS plots of MNP and $CX3CR1^{hi}$ microglia (**E**). Quantification of number of viable MNPs (**F**, n=3, 3, 3, 3, 4 mice from left to right) and $CX3CR1^{hi}$ viable MNPs (**G**, n=3, 3, 3, 3, 4 mice from left to right).

(H, I) Flow cytometry analysis of retinas 4 weeks after initial LPS or PBS injections. Quantification of the percentage of viable MNP ($Ly6G^-/CD11b^+$, **H**) and $CX3CR1^+$ cells of viable MNP ($Ly6G^-/CD11b^+/CX3CR1^+/CD45^+$, **I**) in PBS group (n=4), 1xLPS group (n=4), 4xLPS group (n=4).

(J, K) Flow cytometry analysis of RPE-choroid-sclera 4 weeks after initial LPS or PBS injections. Quantification of the percentage of viable MNP ($Ly6G^-/CD11b^+$, **J**) and $CX3CR1^+$ cells of viable MNP ($Ly6G^-/CD11b^+/CX3CR1^+/CD45^+$, **K**) in PBS group (n=4), 1xLPS group (n=4), 4xLPS group (n=4).

Comparisons between groups were analyzed using one-way ANOVA with Tukey's multiple comparisons test (**B, C, F-K**); $^*P < 0.05$, $^{**}P < 0.01$; error bars represent mean ± SEM.

Supplemental Figure 3: Ablation of retinal myeloid cells in CX3CR1 $^{CreER/+}$:R26 $^{iDTR/+}$ mice following intravitreal diphtheria toxin.

(A, B) Flow cytometry analysis of blood and retina from CX3CR1-fluorescence expressing mice. (**A**) Representative histograms of FITC expression in blood monocytes, neutrophils, B cells, and T cells. (**B**) Representative histograms of FITC expression in mononuclear phagocytes (MNPs).

(C) Flow cytometry analysis of retinas from CX3CR1 $^{CreER/+}$ and CX3CR1 $^{CreER/+}$:R26 $^{iDTR/+}$ mice following intravitreal diphtheria toxin injection. Representative FACS plots of MNPs and $CX3CR1^+$ microglia.

(D, E) Quantification of % viable MNP ($Ly6G^-$ /$CD11b^+$) (**D**) and % $CX3CR1^+$ microglia of viable MNP ($Ly6G^-$ /$CD11b^+$/$CX3CR1^+$ /$CD45^+$) in CX3CR1 $^{CreER/+}$ mice and CX3CR1 $^{CreER/+}$:R26 $^{iDTR/+}$ mice (**E**) (n = 3, 3).
(F) Flow cytometry analysis of RPE-choroid-sclera from CX3CR1 $^{CreER/+}$ and CX3CR1 $^{CreER/+}$:R26 $^{iDTR/+}$ mice following intravitreal diphtheria toxin injection. Representative FACS plots of MNPs and $CX3CR1^+$ microglia.
(G, H) Quantification of % viable MNP ($Ly6G^-$ /$CD11b^+$) (**G**) and % $CX3CR1^+$ microglia of viable MNP ($Ly6G^-$ /$CD11b^+$/$CX3CR1^+$ /$CD45^+$) in CX3CR1 $^{CreER/+}$ mice and CX3CR1 $^{CreER/+}$:R26 $^{iDTR/+}$ mice (**H**) (n = 3, 3).
Comparisons between groups were analyzed using Student's unpaired t-test (**D, E, G, H**); *P < 0.05, ***P < 0.001; error bars represent mean ± SEM.

Supplemental Figure 4: UMAP plot of the gene activity for cell type markers.
(A-F) Gene activity of key marker genes from *Ronning KE* et al. 2019. Classical monocytes **(A)**, inflammatory monocytes **(B)**, circulating monocytes **(C)**, inflammatory macrophages **(D)**, resting microglia **(E)**, and activated microglia **(F)**.

Supplemental Figure 5: Identification of subsets of myeloid cells in $CX3CR1^+$ retinal cells.
(A-E) UMAP plots of gene expression scores using modules (key marker genes from *Ronning KE* et al. 2019**)**. Resting microglia **(A)**, activated microglia **(B)**, circulating monocytes **(C)**, infiltrating monocytes **(D)**, and macrophages **(E)**.
(F, G) Chromatin accessibility in the promoter region of *Tep1* **(F)** and *Atf3* **(G)** in microglia isolated from mice 3 days after CNV induction, preconditioned with either PBS or 4xLPS 1 month before, or Naïve retinas without CNV induction, preconditioned with PBS as analyzed by qPCR. **(H)** Heatmap of top50 cluster-specific gene activity markers. Gene activity scores were then calculated based on the local accessibility of gene regions, and cluster-specific gene markers identified using the following cut-offs: FDR < 0.01 & min.pct > 0.1.
(I) Heatmap of top50 cluster-specific differentially accessible regions (DARs) (each cluster vs all other cells). Cluster-specific DARs were identified using the following cut-offs: FDR < 0.01 & min.pct > 0.1 & absolute average log2 FC > 1.

Supplemental Figure 6：LPS priming induces a sustained altered transcriptional phenotype in myeloid cells.

(A, B) Principal component analysis (PCA) of RNA-seq data of BMDMs isolated from 1xLPS-pretreated, 4xLPS-pretreated, and PBS-pretreated mice without (**A**) and with LPS restimulation (**B**).

(C, D) Heatmaps of the top 60 most differentially expressed (DE) genes of BMDMs isolated from 1xLPS-pretreated, 4xLPS-pretreated, and PBS-pretreated mice without (**C**) and with LPS restimulation (**D**).

(E-H) Quantification of the percentage of total BM monocytes ($Ly6G^-/B220^-/CD3^-/NK1.1^-/CD11b^+/SSC^{low}$, **E**), $Ly6C^{high}$ BM monocytes ($Ly6G^-/B220^-/CD3^-/NK1.1^-/CD11b^+/SSC^{low}/Ly6C^{high}$, **F**), $Ly6C^{inter}$ BM monocytes ($Ly6G^-/B220^-/CD3^-/NK1.1^-/CD11b^+/SSC^{low}/Ly6C^{inter}$, **G**), and $Ly6C^{low}$ BM monocytes ($Ly6G^-/B220^-/CD3^-/NK1.1^-/CD11b^+/SSC^{low}/Ly6C^{low}$, **H**) in PBS group (n=4) and 4xLPS group (n=4).

Comparisons between groups were analyzed using Student's unpaired t-test (**E-H**); *$P < 0.05$; error bars represent mean ± SEM.

Table S1. Primer sequences used for Chlamydia DNA detection.

Gene	Sequence	
Cpn 16S rDNA	F	5’-GGT CTC AAC CCC ATC CGT GTC GG-3’
	R	5’-TGC GGA AAG CTG TAT TTC TAC AGT-3’
mGapdh	F	5’-AAC GGG AAG CTC ACT GGC ATG-3’
	R	5’-TCC ACC ACC CTG TTG CTG TAG-3’

Table S2. Primer sequences used for qPCR.

Gene	Sequence	
mActb	F	5’-GAC GGC CAG GTC ATC ACT ATT G-3’
	R	5’-CCA CAG GAT TCC ATA CCC AAG A-3’
mAif1	F	5’-CCT GAT TGG AGG TGG ATG TCA C-3'
	R	5’-GGC TCA CGA CTG TTT CTT TTT TCC-3'
mAtf3	F	5’-GAG CTG AGA TTC GCC ATC CA-3'
	R	5’-CCG CCT CCT TTT CCT CTC AT-3'
mCol3a1	F	5’-CTG TAA CAT GGA AAC TGG GGA AA-3’
	R	5’-CCA TAG CTG AAC TGA AAA CCA CC-3’
mCxcl1	F	5’-CTGGGATTCACCTCAAGAACATC-3’
	R	5’-CAGGGTCAAGGCAAGCCTC-3'
mIfng	F	5’-GAG GTC AAC AAC CCA CAG GT-3’
	R	5’-GGG ACA ATC TCT TCC CCA CC-3’
mIl1b	F	5’-CTG GTA CAT CAG CAC CTC ACA-3’
	R	5’-GAG CTC CTT AAC ATG CCC TG-3’
mIl6	F	5’-AGA CAA AGC CAG AGT CCT TCA GAG A-3’
	R	5’-GCC ACT CCT TCT GTG ACT CCA GC-3’
mPdgfb	F	5’-CCG GAA CAA ACA CAC CTT CT-3’
	R	5’-TAT CCA TGT AGC CAC CGT CA-3’
mPostn	F	5’-CCT GCC CTT ATA TGC TCT GCT-3’
	R	5’-AAA CAT GGT CAA TAG GCA TCA CT-3’
mTlr4	F	5’-ATG GAA AAG CCT CGA ATC CT-3’
	R	5’-TTT AGG CCC CAG AGT TTT GT-3’
mTnf	F	5’-CCC TCA CAC TCA GAT CAT CTT CT-3’

	R	5’-GCT ACG ACG TGG GCT ACA G-3’
mTgfb1	F	5’-ACG CCT GAG TGG CTG TCT TTT GAC-3’
	R	5’-GGG CTG ATC CCG TTG ATT TCC ACG-3’
mVegfa	F	5’-GCC CTG AGT CAA GAG GAC AG-3’
	R	5’-CTC CTA GGC CCC TCA GAA GT-3’

Table S3. Primer sequences used for chromatin accessibility qPCR.

**Gene**	**Sequence**	
mAtf3	F	5’-CAC CAG GCT CTG AAA CGG AT-3’
	R	5’-TGT CCA ATG GCC AGG GTA TG-3’
mTep1	F	5’-GGG CCA TAG GGT TGC ATC TT-3'
	R	5’-ACA TGT GCA TGC GGT AGA GT-3'

Supplemental Methods

Laser-induced choroidal neovascularization (CNV). Animals were anesthetized intraperitoneally using a mix of 10% ketamine and 4% xylazine (10 µl/g of body weight) and pupils were dilated with Mydriacyl 0.5%. Using an argon laser, we ruptured their Bruch's membrane to induce choroidal neovascularization (CNV) as described previously(1). Each eye received 4 distinct laser burns (400mW, 100µm, 0.05s) distributed equidistantly and following the optic nerve head as central reference. Disruption of the Bruch's membrane was verified via observation of a visible heat bubble at the site of injury. Mice were sacrificed at 3 or 14 days after we induced 4 burns per eye for immunohistochemistry analysis, and 6 burns per eye for the RT - qPCR, FACS analyses, choroidal sprouting assay, and scATAC-seq.

Choroidal neovascularization evaluation. 14 days after choroidal neovascularization (CNV) induction, mice were sedated with isoflurane gas and intracardially perfused with 0.5mL of fluorescein isothiocyanate (FITC)-dextran (average mw: 2,000 kDa) and euthanized. Eye globes were enucleated and fixed in PFA 4% at room temperature for 30 minutes. RPE-choroid-sclera complex was dissected and separated from the neuroretina. After a secondary fixation of 15 min in 4% PFA at room temperature, tissue was incubated for 1 hour in a blocking solution (3% BSA +0.3% Triton X-100) and was followed by an overnight incubation with antibodies. The choroids were stained with rhodamine-labeled *Griffonia (bandeiraea) Simplicifolia* Isolectin I (1:100) and IBA1

(1:250). After several washes with PBS, RPE-choroid-sclera complexes were incubated with secondary fluorochrome-conjugated species-appropriate antibodies for 1 hour. The tissue was then mounted onto a glass slide and imaged using Olympus FluoView FV1000 laser scanning confocal inverted microscope (Olympus Canada, Richmond Hill, ON). For analysis, the Z-stacks were compressed into one image. The area of neovascularization (FITC-dextran positive) and the burn area (Isolectin positive), as well as the number of mononuclear phagocytes (IBA1 positive) were quantified using ImageJ software (Version 1.0; U. S. National Institutes of Health, Bethesda, Maryland, USA).

Chlamydia pneumonia infection. Male C57BL/6J mice, 7 weeks of age, were inoculated intraperitoneally with *C. pneumoniae* (*Cpn*, strain AR-39) 5×10^5 IFU/mouse or mock suspended in sucrose-phosphate-glutamic acid medium. Mice primed 3 days prior to inoculation with thioglycollate broth (total volume, 1.5 mL) injected intraperitoneally.

Chlamydia DNA detection. DNA was extracted from peritoneal macrophages using TRIzol reagent (Cat# 15596026; Invitrogen) according to the manufacturer's instructions. Extracted DNA was used for polymerase chain reaction (PCR) with pairs of primers specific for *Cpn* 16S rDNA and host cellular gapdh (2). The thermal cycling conditions were 95°C for 5 min, followed by 40 cycles of 95°C for 30 s, 60°C for 30 s, and 72°C for 45 s. The amount of amplified DNA of chlamydial 16S rDNA was normalized to that of gapdh DNA. Primer sequences used in this study are listed in the Supplemental Table 1.

Peripheral LPS stimulation. Male, seven-week-old mice were randomly assigned to treatment groups and were injected intraperitoneally (i.p.) with bacterial lipopolysaccharides (LPS, Cat# L6143, Sigma Aldrich) at a daily dose of 0.5mg per kg bodyweight. Animals received either four LPS injections on four consecutive days (4×LPS), a single LPS injection followed by three vehicle injections on the following three days (1×LPS) or four vehicle injections (PBS) as previously reported.

RNA-seq sample preparation and sequencing. Total RNA was isolated from BMDMs using the RNeasy Mini Kit (Qiagen). The mRNA was then purified from 500 ng of total RNA using the Dynabeads mRNA DIRECT Micro Kit (Thermo Fisher Scientific). Whole-transcriptome libraries were prepared using the Ion Total RNA-seq Kit v2. The yield and size distribution of the amplified libraries were assessed with the Agilent 2100 Bioanalyzer using the DNA 1000 Kit. Sequencing was performed on an Ion Proton instrument (Thermo Fisher Scientific).

Transfection of siRNA. BMDMs were transfected with 25nM Atf3 siRNA (ON-TARGETplus Mouse Atf3 siRNA: Target Sequence GAAUGGACGGACACCGGAA) or non-targeting siRNA (ON-TARGETplus Non-targeting Control Pool: Target Sequence UGGUUUACAUGUCGAGUAA, UGGUUUACAUGUUGUGUGA, UGGUUUACAUGUUUUCUGA, UGGUUUACAUGUUUUCCUA) using 0.2% DharmaFECT-4 according to manufacturer instructions. BMDMs were incubated for 24h, and RNA was extracted after 4 hours of LPS stimulation for qPCR analysis.

Choroidal sprouting assay. The ex vivo choroid explant analysis and quantification of microvascular sprouting were performed as described previously (3). Briefly, choroids from *C57Bl/6J* mice were dissected shortly after enucleating eyes. After plating segmented choroids into 24-well tissue culture plates and covering with growth factor reduced Matrigel (BD Biosciences), choroids were cultured in EGM-2 medium. Twenty four hours later, $1x10^5$/ well of BM monocytes from PBS-pretreated, 1xLPS-pretreated, and 4xLPS-pretreated mice, were added to choroidal explant cultures within Transwell inserts (Millicell hanging Cell Culture Insert, PET 0.4µm, Cat# MCHT24H48, Millipore) to investigate the impact of monocytes on microvascular sprouting. Phase contrast photos of individual explants were taken daily using a ZEISS Axio Oberver.Z1 microscope (Zeiss, Oberkochen, Germany). The areas of sprouting were quantified with ImageJ software (Version 1.0; U. S. National Institutes of Health, Bethesda, Maryland, USA).

Bone marrow monocyte isolation. Primary monocytes were isolated from bone marrow of mice. After red blood cell (RBC) lysis using RBC Lysis buffer (Cat# 00-4333-57; eBioscience), the cell pellet was resuspended into isolation buffer (PBS containing 2% FBS and 1 mM EDTA). Monocytes were isolated from the resuspended cell solution using an EasySep™ Mouse Monocyte Isolation Kit (EasySep™ Mouse Monocyte Isolation Kit, STEMCELL Technologies, Cat#19861) according to manufacturer's instructions. Isolated monocytes were resuspended into EGM-2 medium and used for choroidal sprouting assays.

Myeloid cell depletion. Microglia depletion was performed using *CX3CR1*$^{CreER/+}$*:R26*$^{iDTR/+}$ in which the activation of Cre recombinase (under the control of the *Cx3cr1* promoter) can be induced by tamoxifen treatment and leads to surface expression of DTR on CX3CR1-expressing cells. At 6 weeks of age, mice were subjected to daily intraperitoneal injections with tamoxifen diluted in corn oil (4 mg per mouse per day, stock solution at 20mg/ml) for 3 consecutive days. To deplete CX3CR1^{+} cells in *CX3CR1*$^{CreER/+}$*:R26*$^{iDTR/+}$ mice, diphtheria toxin was administered intravitreally (25 ng/1 μl saline per eye) at 11 and 12 weeks of age.

Primary bone marrow-derived macrophages. Bone marrow from both femur and tibia cavities was harvested though bone flushing with PBS supplemented with 10% FBS using a syringe, resuspended and passed through a 70μm strainer. After red blood cell (RBC) lysis using RBC Lysis buffer (Cat# 00-4333-57; eBioscience), bone marrow cells were cultivated in DMEM supplemented with 10% FBS and antibiotics. Macrophage colony-stimulating factor (M-CSF) (20 ng/ml, Cat# PMC2044; Invitrogen) was used to select positively monocytes and differentiate them into macrophages. After 3 days of incubation at 37°C with 5% CO_2, medium containing M-CSF was refreshed. Cells were allowed to differentiate for a total of 6 days before their medium was replaced by complete medium without M-CSF. LPS stimulated cells were stimulated for 24 h with 10 ng/ml LPS. The purity, evaluated by flow cytometry, was usually around 99%.

Serum and tissue endotoxin measurement. The levels of endotoxin in serum, brain, and retina were measured using the Pierce™ LAL Chromogenic Endotoxin Quantitation Kit (Cat # A39552, Thermo Fisher, Waltham, MA, USA), following the manufacturer's instruction. Briefly, 50 μL of diluted protein samples, diluted serum samples, and endotoxin standards were added to each well of a 96-well plate. The plate was warmed to 37 °C, and 50 μL of Amebocyte Lysate Reagent were added to each well and incubated for 10 min following briefly mixing. Then, 100 μL of Chromogenic Substrate Solution were added to each well, and 50 μL of 25% acetic acid were added to wells to stop the reaction following 6-min substrate development. The absorbance at 405 nm was measured using an ELISA plate reader. The concentrations of endotoxin were calculated based on the standard curve created using the absorbance values of the standards.

Evans Blue Permeation Assay. Retinal and brain EB permeation was performed with modifications as previously described (4). EB was injected at 45 mg/kg intravenously, and it was allowed to circulate for 2 hr prior to tissues (retina and brain) extraction. Evans blue permeation was quantified by fluorimetry (620 nm max absorbance, 740 nm minimum absorbance background). Evans blue permeation (measured in μl / [g x hr]) is calculated as (EB [μg] / wet retinal weight [g]) / (plasma EB [μg/μl] x circulation time [hr]). Evans blue permeation was expressed relative to PBS controls.

Transcription analysis by Quantitative PCR. RNA extraction was performed with frozen mouse tissues or BMDMs from in vitro assays using TRIzol reagent (Cat# 15596026;

Invitrogen) and digested with DNase I (Sigma Aldrich; Cat# D4527) following manufacturer instructions to avoid genomic DNA amplification. Total RNA was reverse-transcribed using a 5X All-In-One RT MasterMix (Cat# G590; Applied Biological Materials Inc.) according to the manufacturer's instructions. Gene expression was analyzed using Bright Green2X qPCR Master Mix-Low Rox in an Applied Biosystems 7500 Real-Time PCR System (Thermo Fisher Scientific, Waltham, MA, USA). Primer sequences used in this study are listed in the Supplemental Table 2. Analysis of expression was followed using the ΔΔCT method. *Actb* expression was used as the reference housekeeping gene. Statistical analysis was performed on ΔΔCT values, and data was represented as the expression of the target genes normalized to *Actb* (folds of increase).

Fluorescence-Activated Cell Sorting (FACS) on retina and RPE-choroid-sclera complexes.

Retinas and RPE-choroid-sclera complexes were cut into small pieces and homogenized in a solution of 750U/mL DNAse I (Cat# D4527, Sigma-Aldrich) and 0.5mg/mL of collagenase D (Cat# 11088866001, Roche) for 20 minutes at 37°C. Homogenates were filtered through a 70µm cell strainer, counted and resuspended in PBS +3% FBS. Viability of the cells was checked by 7-ADD (Cat# 420404: Biolegend) staining for 20 min at room temperature. After incubation with LEAF purified anti-mouse CD16/32 (Cat# 101310; BioLegend) for 10 minutes at 4ºC to block FC receptors, cells were incubated for 25 minutes at 4ºC with the following antibodies:

BV711 anti-mouse/human CD11b (Cat# 101242; BioLegend), PE anti-mouse CX3CR1 (Cat# FAB5825P; R&D), APC anti-mouse CD45.2 (Cat# 109814; BioLegend), APC/Cy7

anti-mouse Ly-6G (Cat# 127624; BioLegend) and PE/Cy7 anti-mouse F4/80 (Cat# 123114; BioLegend). Fluorescence-activated cell sorting (FACS) was performed on a BD LSR FortessaTM X-20 cell analyzer, and data were analyzed using FlowJo software (Version 10.2; FlowJo, Ashland, OR, USA).

Fluorescence-Activated Cell Sorting (FACS) on BMDMs. BMDMs were collected in PBS through scraping. Viability of the cells was verified by Zombie Aqua (423101: BioLegend) staining for 15 min at room temperature. Cells were incubated with LEAF‐purified anti‐mouse CD16/32 (101310; BioLegend) for 10 min at 4°C to block Fc receptors, and were then incubated for 25 min at 4°C with the following antibodies: BV711 anti‐mouse/human CD11b (101242; BioLegend), PE anti‐mouse F4/80 (123110; BioLegend), APC anti‐mouse CD64 (139305; BioLegend), APC/Cy7 anti‐mouse Ly‐6G (127624; BioLegend), BV785 anti‐mouse CD11c (117335; BioLegend), and PE/Cy7 anti‐mouse CD206 (141719; BioLegend). Fluorescence-activated cell sorting (FACS) was performed on a BD LSR FortessaTM X-20 cell analyzer, and data were analyzed using FlowJo software (Version 10.2; FlowJo, Ashland, OR, USA).

Fluorescence-Activated Cell Sorting (FACS) on peripheral blood. For monocyte analysis, blood was collected using cardiac puncture. After RBC lysis, cells were counted and resuspended in PBS+3% FBS. Viability of the cells was checked by 7-ADD (Cat# 420404: Biolegend) staining for 20 min at room temperature. Cells were incubated with LEAF‐purified anti‐mouse CD16/32 (101310; BioLegend) for 10 min at 4°C to block Fc

receptors, and were then incubated for 25 min at 4°C with the following antibodies: APC anti-mouse CD45.2 (Cat# 109814; BioLegend), BV711 anti‐mouse/human CD11b (101242; BioLegend), APC/Cy7 anti‐mouse Ly‐6G (127624; BioLegend), A700 anti-mouse Ly 6C (128024; BioLegend), PE/Cy7 anti-mouse CD115(135523; BioLegend). Fluorescence-activated cell sorting (FACS) was performed on a BD LSR FortessaTM X-20 cell analyzer, and data were analyzed using FlowJo software (Version 10.2; FlowJo, Ashland, OR, USA). Monocytes were gated as CD115+CD11b+ Ly6C+CD45.2+Ly6G- population.

Western blot analysis. For assessment of BMDM protein levels, we collected BMDMs by scraping the cells in 1× RIPA on ice. Protein concentration was assessed by bicinchoninic acid (BCA) assay (Sigma‐Aldrich), and 20 μg protein was loaded for each condition by standard SDS–PAGE technique. Anti‐ATF3 (ab207434, Abcam) (1:1,000), Anti-P-cJun (Cat# 3270, Cell Signaling) (1:1,000) and Anti‐P-NF‐κB p65 (Cat# 3031, Cell Signaling) (1:1,000) antibodies were used.

Chromatin accessibility assay. Mouse-specific chromatin accessibility was analyzed by nuclease-dependent chromatin degradation via qPCR using EpiQuik chromatin accessibility assay kit (Epigentek, Cat# P-1047). Chromatin DNA was isolated from $4x10^5$ cells and treated with or without a nuclease mix (Nse) following the manufacturer's instructions. DNA was purified with RNase 1 and amplified using real-time PCR targeting specific promoter regions. The fold enrichment was calculated using a ratio of

amplification efficiency of Nse-treated DNA sample over that of no-Nse control sample: Fold enrichment = $2^{(\text{Nse CT} - \text{no-Nse CT})}$ x 100%.

The primers sequences used for chromatin accessibility are listed in Table S3.

scATAC-Sequencing sample preparation . FACS-sorted GFP+ cells from CX3CR1$^{GFP/+}$ mice were used for scATAC-seq and nuclei isolation was performed according to the protocol of 10x Genomics. Briefly, isolated cells were centrifuged at 300x*g* for 5 min at 4°C and then resuspended in ice-cold PBS+0.04% BSA. The cell lysis was performed for 4 minutes on ice by adding 45μl lysis buffer (10 mM Tris-HCl, pH 7.4, 10 mM NaCl, 3 mM MgCl2, 0.1% Tween-20, 0.1% Nonidet P-40, 0.01% Digitonin, and 1% BSA). After lysis, 50μl ice-cold wash buffer (10 mM Tris-HCl, pH 7.4, 10 mM NaCl, 3 mM MgCl2, 0.1% Tween-20, and 1% BSA) were added and then centrifuged at 500 × g for 5 min at 4 °C. After one more wash, the washed nuclei were counted and used for the transposase reaction.

After removing the supernatant, the pellet was resuspended in 7 ml 1x Nuclei Buffer (10x Genomics) and 2 ml of the nuclei suspension was taken for evaluation (nuclei quality control and determining nuclei concentration). Nuclei suspension was then used according to the Chromium Single Cell ATAC Reagent Kits protocol (10x Genomics). The calculated volume of the nuclei suspension (targeted nuclei recovery is 1600 nuclei/sample) was mixed with the Transposition mix and incubated for 1 h at 37 ℃. After finishing the transposition, the transposed nuclei were mixed with a barcoding mix and loaded into a 10x chip H (10x Genomics, Chromium Next GEM Chip H Single Cell Kit v1.1; Chromium Next GEM Single Cell ATAC Library & Gel Bead Kit v1.1) together

with barcoded beads and partitioning oil and encapsulated using the Chromium controller. The gel emulsion was transferred into a PCR tube to perform 12 cycles of amplification in a thermocycler. The gel emulsion containing barcoded DNA was broken, purified (10x Genomics, Dynabeads MyOne SILANE) and subjected to a final index PCR for 11 cycles. After size selection (0.4x/1.2x volume of beads) (Beckman Coulter SPRIselect), the library was examined on a fragment analyzer (Agilent, NGS High Sensitivity Fragment Analysis Kit) for its quality and quantity and sequenced on an Illumina NovaSeq 6000 SP PE100. More information on the library prep can be found at the site https://www.10xgenomics.com/resources/support-documentation

Bioinformatic analysis were performed at the Bioinformatics core facility of the Institut de Recherches Cliniques de Montréal. Sequencing reaction was performed at the sequencing platform of Centre d'expertise et de services Génome Québec.

RNA-seq analysis. Raw data was were analyzed using Torrent Suite Software 5.12.0 (Thermo Fisher Scientific). Differential expression analysis was performed with DESeq2 and gene set enrichment analysis GSEA was conducted using GSEA v3.0 software provided by Broad Institute of Massachusetts Institute of Technology and Harvard University.

scATAC-Sequencing analysis. FASTQ files were processed using Cellranger-atac (v1.2.0). Sequencing reads for all 3 samples were aligned to mm10 (cellranger-atac reference v1.2.0, mm10) and pooled together while normalizing libraries for sequencing depth. Aggregated

files were then used for quality control, filtering and downstream analyses in Signac v1.1.1(5). Only cells with nucleosome signal < 4, transcription start site enrichment > 2, alignment to blacklisted regions < 5%, number of reads in peaks < 40 000 and read fractions in peaks >20% were kept, resulting in a total of 2244 cells (835 PBS, 588 Ø laser-burn, and 821 4xLPS).

Normalization and dimensionality reduction were performed using latent semantic indexing (LSI), i.e term frequency–inverse document frequency (TF-IDF) normalization, followed by singular value decomposition (SVD) to generate a lower dimensional space(6). Only LSI dimensions 2 to 10 were kept (the first dimension was excluded since it was highly correlated with read depth). For visualization, UMAP was also applied.

Clusters were identified using the Leiden algorithm with a resolution of 0.4 and the differential accessible regions for each cluster (or comparing samples) through logistic regression with the total number of fragments as a latent variable to control for sequencing depth (or using the Wilcoxon Rank Sum test). Gene activity scores were then calculated based on the local accessibility of gene regions, and cluster-specific gene markers identified using the following cut-offs: FDR < 0.01 & min.pct > 0.1 and presented in a heatmap. C1, C2 and C3 specific DARs were defined using adjusted p-value <0.05 and min.pct>0.1 giving respectively 134/406, 276/547, 51/202 more closed/opened regions. DARs between C2 PBS and C3 4xLPS were defined with adjusted p-value <0.05 giving 51 DARs.

Clusters identified as microglia cells (using gene activity scores) were subsetted and used for identification of cis-co-accessible networks using Cicero packages(7).

References

1. Lambert V, Lecomte J, Hansen S, Blacher S, Gonzalez ML, Struman I, Sounni NE, Rozet E, de Tullio P, Foidart JM, et al. Laser-induced choroidal neovascularization model to study age-related macular degeneration in mice. *Nat Protoc.* 2013;8(11):2197-211.

2. Matsuo J, Sakai K, Okubo T, and Yamaguchi H. Chlamydia pneumoniae enhances Interleukin 8 (IL-8) production with reduced azithromycin sensitivity under hypoxia. *APMIS.* 2019;127(3):131-8.

3. Shao Z, Friedlander M, Hurst CG, Cui Z, Pei DT, Evans LP, Juan AM, Tahiri H, Duhamel F, Chen J, et al. Choroid sprouting assay: an ex vivo model of microvascular angiogenesis. *PLoS One.* 2013;8(7):e69552.

4. Cerani A, Tetreault N, Menard C, Lapalme E, Patel C, Sitaras N, Beaudoin F, Leboeuf D, De Guire V, Binet F, et al. Neuron-derived semaphorin 3A is an early inducer of vascular permeability in diabetic retinopathy via neuropilin-1. *Cell Metab.* 2013;18(4):505-18.

5. Stuart T, Srivastava A, Madad S, Lareau CA, and Satija R. Single-cell chromatin state analysis with Signac. *Nat Methods.* 2021;18(11):1333-41.

6. Cusanovich DA, Daza R, Adey A, Pliner HA, Christiansen L, Gunderson KL, Steemers FJ, Trapnell C, and Shendure J. Multiplex single cell profiling of chromatin accessibility by combinatorial cellular indexing. *Science.* 2015;348(6237):910-4.

7. Pliner HA, Packer JS, McFaline-Figueroa JL, Cusanovich DA, Daza RM, Aghamirzaie D, Srivatsan S, Qiu X, Jackson D, Minkina A, et al. Cicero Predicts cis-Regulatory DNA Interactions from Single-Cell Chromatin Accessibility Data. *Mol Cell.* 2018;71(5):858-71 e8.
